# A novel role for interleukin 32 in cholestasis

**DOI:** 10.1002/ctm2.594

**Published:** 2021-11-23

**Authors:** Xiaoxun Zhang, Ling Li, Nan Zhao, Qiong Pan, Liangjun Zhang, Qiaoling Xie, Xuan Li, Min Liao, Qiao Li, Xinglin Huang, Sheng Chen, Jianwei Li, Huaizhi Wang, Xuequan Huang, Shijun Fan, Yunxia Wang, Man Li, Jin Chai

**Affiliations:** ^1^ Cholestatic Liver Diseases Center and Department of Gastroenterology Southwest Hospital Third Military Medical University (Army Medical University) Chongqing China; ^2^ Department of Pediatrics Southwest Hospital Third Military Medical University (Army Medical University) Chongqing China; ^3^ Institute of Hepatobiliary Surgery Southwest Hospital Third Military Medical University (Army Medical University) Chongqing China; ^4^ Institute of Hepatopancreatobiliary Surgery Chongqing General Hospital University of Chinese Academy of Sciences Chongqing China; ^5^ Department of Radiology Southwest Hospital Third Military Medical University (Army Medical University) Chongqing China; ^6^ Medical Research Center Southwest Hospital Third Military Medical University (Army Medical University) Chongqing China; ^7^ Department of Clinical Laboratory Medicine Southwest Hospital Third Military Medical University (Army Medical University) Chongqing China; ^8^ Department of Internal Medicine and Liver Center Yale University School of Medicine New Haven Connecticut USA

Dear editor,

We report here, for the first time, that interleukin 32 (IL32) expression is elevated in the liver of patients with obstructive cholestasis, and its upregulation ameliorates cholestatic liver injury by repressing bile acid (BA) synthesis and the inflammatory response.

Cholestasis is characterized by excessive accumulation of intrahepatic BA that causes liver injury and inflammation.^1^, ^2^ IL32, a predominantly intracellular proinflammatory mediator, is involved in infectious diseases and cancers,^3^, ^4^, ^5^, ^6^, ^7^ but at present, its role in cholestasis remains unknown. Here, we demonstrated that hepatic IL32 mRNA and protein levels were markedly increased (Figure 1A‐C) in obstructive cholestatic patients (Table S1), as well as in patients with other types of cholestasis, including primary biliary cholangitis (PBC) and PBC plus autoimmune hepatitis (Figure S1), when compared to their controls. Interestingly, hepatic IL32 mRNA levels were negatively correlated with the serum levels of biochemical markers of liver injury, including ALT, AST, ALP and GGT in patients with obstructive cholestasis (*p *< 0.05, Figure 1D), implying a protective role of hepatic IL32 in cholestasis.

**FIGURE 1:**
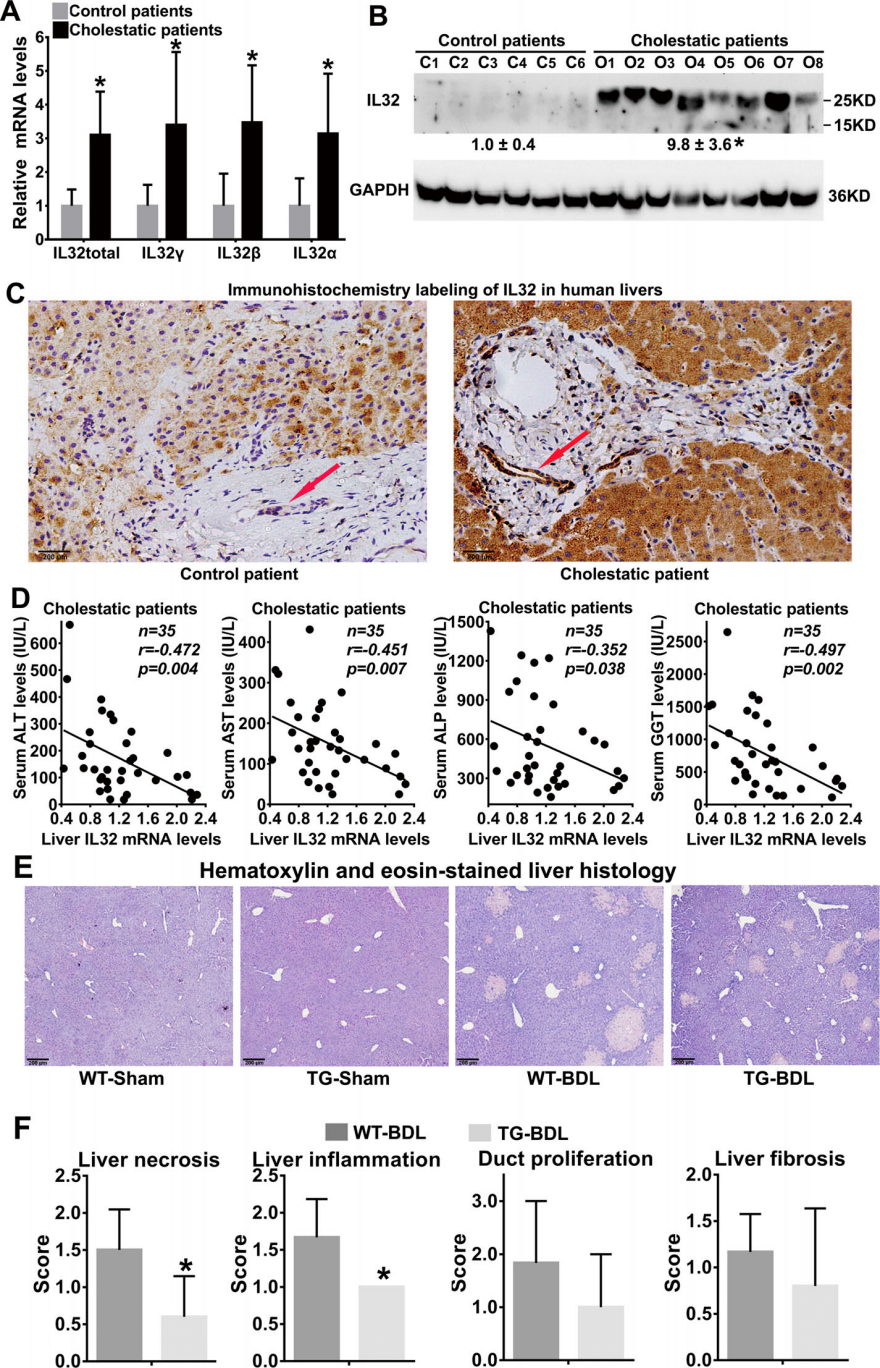
Hepatic interleukin‐32 (IL32) expression was markedly increased in obstructive cholestatic patients, and less liver necrosis and inflammation were observed in the *hIL32γLTg*‐BDL group. (A) The mRNA expression of total IL32 and its major isoforms in the liver tissues of patients with or without obstructive cholestasis. (B) Representative western blots for IL32 protein expression in the liver tissues of patients with or without obstructive cholestasis. *C1‐6*, controls; *O1‐8*, obstructive cholestasis liver tissues; obstructive cholestatic group versus control group, *p < *0.05*. (C) Immunohistochemistry (IHC) labeling of IL32 protein in the liver of a control patient (left) and a patient with obstructive cholestasis (right). IL32 expression in cholestatic hepatocytes was increased. Interestingly, increased expression of IL32 in bile duct epithelial cells was also observed (arrows). (D) Hepatic mRNA levels of IL32 showed a negative correlation with serum levels of ALT, AST, ALP, and GGT in patients with obstructive cholestasis (*n* = 35, *p *< 0.05). (E) Hematoxylin and eosin‐stained (H&E) liver histology and (F) scores for liver injury, including necrosis, inflammation, bile duct proliferation, and fibrosis, as assessed by pathologists blinded to the experimental conditions. Sham, sham operation; BDL, bile duct‐ligation; WT, wild‐type; TG; liver‐specific human *IL32γ* transgenic (*hIL32γLTg*) mice. *p < 0.05 versus WT BDL group

It has been reported that human *IL32*‐overexpressing transgenic mouse models were used to investigate the function of IL32 in synovial joints, atherosclerosis and other diseases.^6^, ^7^ To elucidate the role of hepatic IL32 in cholestasis, we generated liver‐specific human *IL32γ‐*transgenic (hIL32γLTg) mice (Figure S2A‐C) and then induced cholestasis with bile duct‐ligation (BDL) or 1%‐cholic‐acid (CA)‐feeding. In BDL‐mice, liver histologic assessment indicated that liver necrosis and inflammation were significantly lower in the *hIL32γLTg*‐BDL group with less bile duct proliferation and fibrosis compared with the WT‐BDL group (Figure 1E,F). Furthermore, the results of serum biochemistry tests were in agreement with the above observations (Table S2). Moreover, the levels of hepatic BA and 7‐α‐C4 were significantly lower in the *hIL32γLTg*‐BDL mice than in the WT‐BDL mice (Table S2), indicating that hepatic IL32 represses BA synthesis. Similar results were also obtained on the 14‐day following 1%CA‐feeding in *hIL32γLTg* mice (Table S3). Together, these data indicate that hepatic IL32 can ameliorate cholestatic liver injury by repressing BA synthesis and liver inflammation.

To reveal how IL32 represses BA synthesis in cholestasis, we assessed the expression of major genes involved in the biosynthesis and transportation of BA. As shown in Figure 2A‐C, cholesterol‐7‐alpha‐hydroxy‐lase (Cyp7a1), the key rate‐limiting enzyme in the biosynthesis of BA, was markedly downregulated while detoxification enzymes Cyp2b10, Ugt1a1 and Sult2a1/2 were significantly increased in *hIL32γLTg* mice compared with WT mice after BDL. Among hepatic BA transporters, we observed significantly increased expression of organic‐solute‐transporter (Ost) α/β, but decreased expression of organic‐anion‐transporting‐polypeptide‐1b2 (Oatp1b2) and no changes in other BA transporter levels (Figure 2A‐C). Furthermore, there were no significant changes in the mRNA levels of fibroblast‐growth‐factor‐receptor (FGFR4) and nuclear receptor farnesoid‐X‐receptor (FXR/NR1H4) in the *hIL32γLTg*‐BDL mice compared with the WT‐BDL mice, while the nuclear FXR protein level was significantly elevated along with increased short‐heterodimer‐partner (SHP) mRNA and nuclear protein levels (Figure 2D‐F). Chromatin immunoprecipitation (ChIP) assays further revealed that the binding activity of FXR to the *SHP* promoter^8^, ^9^ was significantly increased in *hIL32γLTg* compared with WT mice after BDL (Figure 2G). These data imply that hepatic IL32 could increase FXR activation through post‐translational regulation in cholestasis (Figure 2 and Figure S3). A recent report indicated that β‐catenin suppression promotes FXR nuclear translocation through FXR/β‐catenin protein complex disassociation.^9^ Indeed, we also observed downregulation of β‐catenin and FXR nuclear translocation in *hIL32γLTg*‐BDL mice (Figure S4A‐C). Co‐immunoprecipitation data showed that less β‐catenin was detected in the precipitated‐FXR complexes in *hIL32γLTg*‐BDL mice compared with WT‐BDL mice (Figure S4D). These data suggest that IL32 promoted FXR/β‐catenin protein complex dissociation and thereby increased FXR nuclear translocation. These results were validated in human obstructive cholestatic livers (Figure S5A‐C).

**FIGURE 2:**
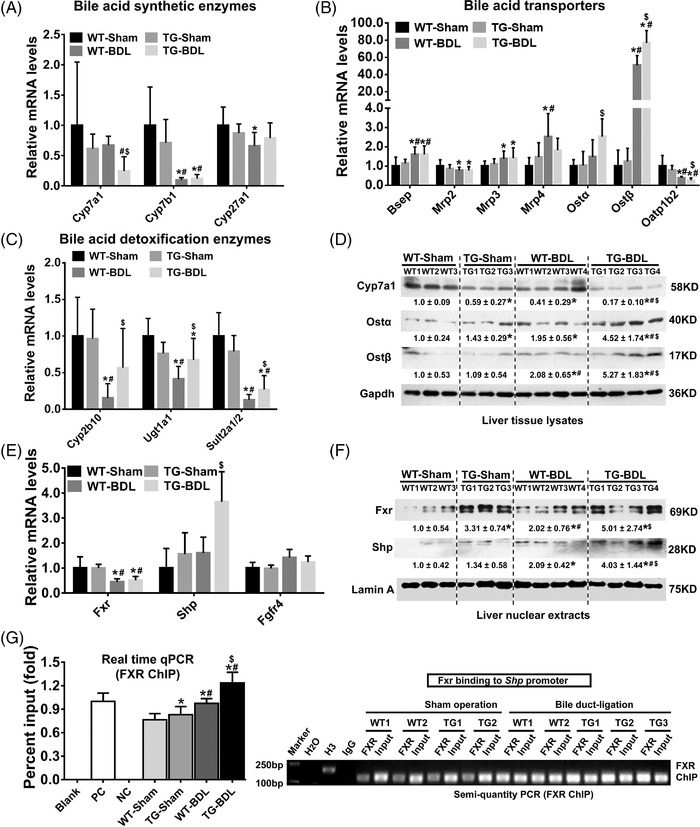
Overexpression of hepatic interleukin‐32 (IL32) increased farnesoid‐X‐receptor (FXR) activation and repressed Cyp7a1 expression in cholestasis. Hepatic mRNA levels of (A) bile acid (BA) synthetic enzymes Cyp7a1, Cyp7b1, and Cyp27a1, (B) BA transporters Bsep, Mrp2, Mrp3, Mrp4, Ostα, Ostβ and Oatp1b2, as well as (C) detoxification enzymes Cyp2b10, Ugt1a1 and Sult2a1/2. (D) Representative western blots for Cyp7a1, Ostα and Ostβ protein expression. (E) Hepatic mRNA and (F) nuclear protein levels of FXR, SHP and/or Fgf4. (G) ChIP assay results (left, quantitative real‐time polymerase chain reaction (RT‐qPCR); right, semi‐quantitative PCR) demonstrated that increased FXR binding activity to its response elements (FXR ChIP) in the *SHP* promoter was observed in TG‐bile duct‐ligation (BDL) mice compared with WT‐BDL mice. WT‐Sham, sham operation WT group (*n* = 7); TG‐Sham, sham operation *hIL32γLTg* group (*n* = 8); WT‐BDL, bile duct‐ligated wild‐type group (*n* = 12); TG‐BDL, bile duct‐ligated *hIL32γLTg* group (*n* = 11). * *p <* 0.05 versus WT‐Sham group; **#** *p <* 0.05 versus TG‐Sham group; **$** *p <* 0.05 versus WT‐BDL group

Next, we investigated how IL32 suppressed the liver inflammatory response in cholestasis. Fluorescence‐activated‐cell‐sorting, quantitative real‐time polymerase chain reaction (RT‐qPCR), western‐blotting and IHC analyses demonstrated that hepatic neutrophil and CD8+‐T‐cell infiltration were significantly lower in *hIL32γLTg*‐BDL mice compared with WT‐BDL mice (Figure S6A‐C and S7A,B). Accordingly, RT‐qPCR analysis revealed that hepatic mRNA levels of chemokines Cxcl5, Cxcl10 and Ccl2, as well as their receptors Cxcr2, Cxcr3 and Ccr2, were markedly decreased in *hIL32γLTg*‐BDL mice compared with WT‐BDL mice (Figures S6D,E and S8A‐C). These data indicate that IL32 reduced neutrophil and CD8+‐T‐cell infiltration in cholestatic livers by repressing Cxcl5, Cxlc10 and Ccl2 expression. In addition, our previous^10^ and present data demonstrate that conjugate BAs, including TCA, TCDCA, GCA and GCDCA, stimulated the expression of chemokines Cxcl5, Cxcl10 and Ccl2 in primary WT mouse hepatocytes (Figure 3A,B and Figure S9A). However, these effects were abrogated in primary *hIL32γLTg* mouse hepatocytes (Figure 3A,B and Figure S9A), suggesting that IL32 represses BA‐stimulated chemokine expression. Furthermore, IL32 overexpression markedly repressed the expression of these chemokines, c‐Jun and phospho–c‐Jun N‐terminal kinase (JNK) in BA‐stimulated primary hepatocytes and BDL livers of *hIL32γLTg* mice compared to WT mice (Figure 3C,D). However, these effects were diminished in the presence of Anisomycin, an agonist of JNK/MAPK signaling (Figure 3E). Moreover, ChIP qPCR assays demonstrated that the binding activities of c‐Jun to the *Cxcl5, Cxcl10* or *Ccl2* promoters (c‐Jun ChIP3 site located *Cxcl5* ‐924 to ‐918, ChIP4 *Cxcl10* ‐765 to ‐759 and ChIP6 *Ccl2* ‐21 to ‐15) were significantly decreased in the liver tissues of *hIL32γLTg*‐BDL mice compared to WT‐BDL mice (Figure 3F and Figure S9B,C). These data supported that hepatic IL32 repressed BA‐induced expression of chemokines Cxcl5, Cxcl10 and Ccl2 by inhibiting the activation of the JNK/MAPK signaling pathways. Similar results were also observed in the liver of patients with obstructive cholestasis (Figure S10A‐C).

**FIGURE 3:**
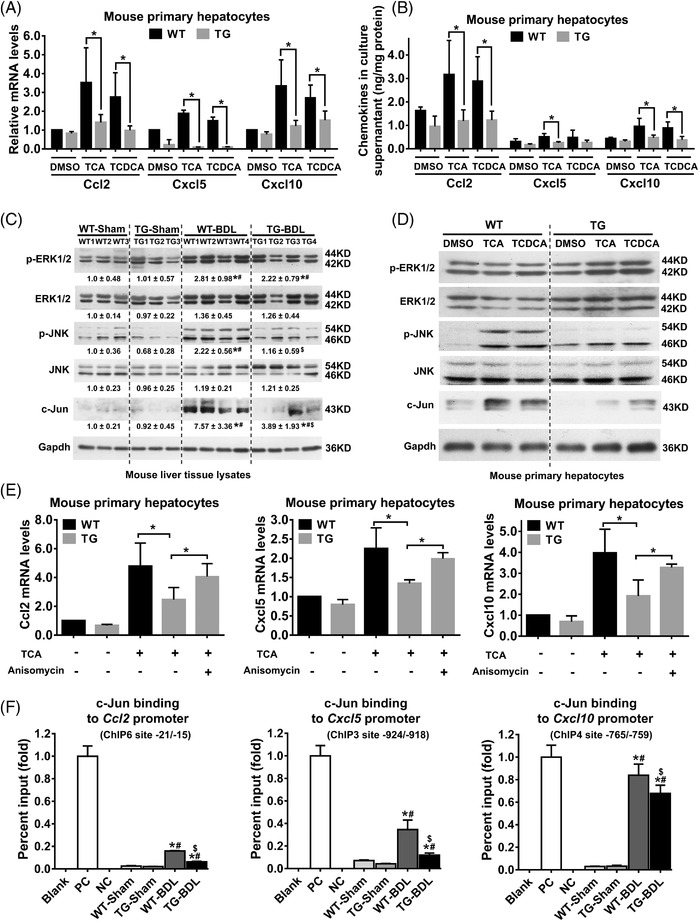
Overexpression of hepatic IL32 repressed bile acid (BA)‐induced chemokine Cxcl5, Cxcl10 and Ccl2 expression in cholestatic hepatocytes by inhibiting JNK/MAPK signaling activation. (A) mRNA levels of chemokines Cxcl5, Cxcl10 and Ccl2 expression in WT and *hIL32γLTg* primary mouse hepatocytes after conjugate BA TCA and TCDCA treatment. *p < *0.05*, *n* = 3. (B) Protein levels of Cxcl5, Cxcl10 and Ccl2 in the culture supernatant of BA‐treated WT and TG primary mouse hepatocytes. *p < *0.05*, *n* = 3. (C) Representative western blots and corresponding densitometry of phospho‐extracellular signal–regulated kinase (ERK), ERK, phospho‐c‐Jun N‐terminal kinase (JNK), JNK, and c‐Jun from mouse liver tissue lysates. WT‐Sham, sham operation WT group (*n* = 7); TG‐Sham, sham operation *hIL32γLTg* group (*n* = 8); WT‐bile duct‐ligation (BDL), bile duct‐ligated wild‐type group (*n* = 12); TG‐BDL, bile duct‐ligated *hIL32γLTg* group (*n* = 11). * *p <* 0.05 versus WT‐Sham group; **#** *p <* 0.05 versus TG‐Sham group; **$** *p <* 0.05 versus WT‐BDL group. (D) Conjugate BAs TCA and TCDCA (25 μM) induced phosphorylation of phospho‐JNK and expression of c‐Jun in WT primary mouse hepatocytes, whereas the induction was diminished in *hIL32γLTg* primary mouse hepatocytes. (E) BAs (e.g., TCA, TCDCA, GCA and GCDCA) significantly induced mRNA expression of chemokines Ccl2, Cxcl5 and Cxcl10 in WT primary mouse hepatocytes (Figure S9A). However, this effect was not observed in *hIL32γLTg* primary mouse hepatocytes, but could be recovered in the presence of a JNK/MAPK signaling agonist (5 μM Anisomycin). (F) ChIP assay results revealed that binding activities of c‐Jun to the *Ccl2* (ChIP6 site ‐21/‐15)*, Cxcl5* (ChIP3 site ‐924/‐918) and *Cxcl10* promoter (ChIP4 site ‐765/‐759) were significantly decreased in TG‐BDL mouse livers, compared to WT‐BDL mouse livers. * *p <* 0.05 versus WT‐Sham group; **#** *p <* 0.05 versus TG‐Sham group; **$** *p <* 0.05 versus WT‐BDL group

Finally, we explored the potential of IL32 gene therapy for the treatment of PSC in *Abcb4‐*KO mice (Figure S11), a model of PSC. Strikingly, serum ALT, AST and ALP levels, as well as total hepatic BA, 7‐α‐C4 and Cyp7a1 protein levels, were significantly lower (Table 1 and Figure S12A) in *Abcb4*‐KO mice with AAV8‐*hIL32γ* injection than in *Abcb4*‐KO mice with AAV8‐control (*CTR)* injection (Figures S13 and S14). Furthermore, hepatic Ccl2, Cxcl5, Cxcl10 and Ptprc levels were also markedly decreased (Figure S12B). These results support that hepatic IL32 overexpression attenuated cholestatic liver injury by repressing intrahepatic BA accumulation and the liver inflammatory response. Moreover, RT‐qPCR, Sirius Red, Masson Trichrome, H&E staining and IHC labeling of CK19 analyses revealed that overexpression of IL32 also could be beneficial for the treatment of cholestatic liver fibrosis (Figures S12C and S15).

**TABLE 1:** Serum biochemistry in Abcb4‐KO mice with AAV8‐hIL32γ injection

	Saline	AAV8‐*CTR*	AAV8‐*hIL32γ*
	WT (*n* = 7)	*Abcb4*‐KO (*n* = 6)	*Abcb4*‐KO (*n* = 7)
Gender (male/female)	4 / 3	3 / 3	4 / 3
Serum ALT *(IU/L)*	45.18 ± 8.78	171.87 ± 49.88*	88.25 ± 25.48*, ^#^
Serum AST *(IU/L)*	141.43 ± 16.35	297.53 ± 47.50*	169.49 ± 30.10*, ^, #^
Serum ALP *(IU/L)*	64.11 ± 16.82	168.96 ± 106.32*	67.13 ± 25.81^#^
Serum TBA *(μmol/L)*	1.13 ± 0.64	11.88 ± 9.60*	11.42 ± 14.13
Serum TBIL *(μmol/L)*	2.94 ± 2.47	0.71 ± 0.27	4.45 ± 4.63
Serum DBIL *(μmol/L)*	4.17 ± 1.16	0.21 ± 0.0	7.29 ± 2.07
Liver tissue BAs *(μmol/kg of liver)*	150.81 ± 46.48	229.90 ± 35.37*	168.67 ± 28.68^#^
Liver 7‐α‐C4 (*ng/g of liver*)	Not detected	95.56 ± 76.71	45.43 ± 19.38

*Note*: Values are means ± SD.

Abbreviations: ALP, alkaline phosphatase; ALT, alanine aminotransferase; AST, aspartate aminotransferase; CTR, control; DBIL, direct bilirubin.; TBA, total bile salts; TBIL, total bilirubin; WT, wild‐type.

* *p < *0.05 versus WT mice with saline injection.

^#^ *p <* 0.05 versus *Abcb4*‐KO mice with AAV8‐*CTR* injection.

In summary, hepatic IL32 acts as a novel repressor of BA synthesis and the liver inflammatory response in cholestasis (Figure S16). These findings advance our understanding of the molecular mechanisms of cholestasis and provide a promising treatment strategy for human cholestasis.

## CONFLICT OF INTEREST

The authors have no conflict of interest to disclose.

## FUNDING INFORMATION

National Natural Science Foundation of China, Grant Numbers: 81770583, 82000545 and 81922012; Natural Science Foundation of Chongqing, Grant Number: cstc2016jcyjA0149; Southwest Hospital Science Foundation, Grant Numbers: 2017YQRC‐01 and XZ‐2019‐505‐001.

## Supporting information

Supporting Information
