# Supplementary material for: A novel role for interleukin 32 in cholestasis
Source: Clin Transl Med. 2021 Nov 23;11(11):e594. doi: 10.1002/ctm2.594 (PMC8611182; doi:10.1002/ctm2.594)
Supplement: Supplementary file 1 — Supporting Information [file CTM2-11-e594-s001.docx]

***Supporting documents***

**A novel role for interleukin 32 in cholestasis**

Xiaoxun Zhang^1^*, Ling Li^1^*, Nan Zhao^1^*, Qiong Pan^1^, Liangjun Zhang^1^, Qiaoling Xie^1^, Xuan Li^1^, Min Liao^1^, Qiao Li^1^, Xinglin Huang^1^, Sheng Chen^2^, Jianwei Li^3^, Huaizhi Wang^3, 4^, Xuequan Huang^5^, Shijun Fan^6^, Yunxia Wang^7^, Man Li^8^, and Jin Chai^1#^

^1^Cholestatic Liver Diseases Center and Department of Gastroenterology, Southwest Hospital, Third Military Medical University (Army Medical University), Chongqing 400038, China

^2^Department of Pediatrics, Southwest Hospital, Third Military Medical University (Army Medical University), Chongqing 400038, China

^3^Institute of Hepatobiliary Surgery, Southwest Hospital, Third Military Medical University (Army Medical University), Chongqing 400038, China

^4^Institute of Hepatopancreatobiliary Surgery, Chongqing General Hospital, University of Chinese Academy of Sciences, Chongqing 400038, China

^5^Department of Radiology, Southwest Hospital, Third Military Medical University (Army Medical University), Chongqing 400038, China

^6^Medical Research Center, Southwest Hospital, Third Military Medical University (Army Medical University), Chongqing 400038, China

^7^Department of Clinical Laboratory Medicine, Southwest Hospital, Third Military Medical University (Army Medical University), Chongqing 400038, China

^8^Department of Internal Medicine and Liver Center, Yale University School of Medicine, 333 Cedar Street, New Haven, CT 06520, USA

*These authors contributed equally to this study.

**Contact Information:**

#Jin Chai, M.D., Ph.D., Professor of Gastroenterology and Hepatology, Director of Cholestatic Liver Diseases Center (CLDC) and Department of Gastroenterology, Southwest Hospital, Chongqing, 400038, China. Tel: 86-23-68765191; Fax: 86-23-65410853; E-mail: [jin.chai@cldcsw.org](mailto:jin.chai@cldcsw.org). Jin Chai’ Research Team Website: www.cldcsw.org.

**Suppl. Materials and Methods**

***Patients and liver sample collection***

Patients were recruited from the Institute of Hepatobiliary Surgery in the Southwest Hospital. Patients underwent pancreastoduodenectomy (PD) resection with curative intent. Cholestatic liver samples (n=35) were obtained from patients suspected of having a pancreatic or periampullary malignancy. All patients had obstructive cholestasis with jaundice caused by periampullary tumor growth at initial presentation and underwent surgery within 1 week without preoperative biliary drainage. Control liver samples were also obtained from patients undergoing resection of liver metastases without cholestasis, as described previously [Suppl. Ref. 1] (n=22; 3 neuroendocrine tumors, 7 colorectal, 7 colonic, and 5 rectal metastases). In addition, liver biopsy samples were obtained from patients with PBC (n=10), autoimmune hepatitis (AIH, n=3), and PBC plus AIH (n=2). The collected liver samples were immediately cut into small pieces and fixed in 4% paraformaldehyde or stored in liquid nitrogen. The characteristics of the included patients are summarized in Table.S1.

***Chemicals and recombinant cytokine***

Bile acids (BAs), including taurochenodeoxycholate acid (TCDCA), glycochenodexycholate acid (GCDCA), taurocholic acid (TCA), and glycocholate acid (GCA) as well as dimethyl sulfoxide (DMSO) [10, Suppl. Ref. 1] were purchased from Sigma-Aldrich Chemical Co. (St Louis, MO, USA). Anisomycin (Cat#HY-18982), an agonist of JNK/MAPK signaling, was purchased from MedChemExpress CO., Ltd (Monmouth Junction, NJ, USA).

The study protocol was reviewed and approved by the Institutional Ethics Review Board of the Southwest Hospital (Chongqing, China). Corresponding written informed consent was obtained from all patients prior to the study. The study involving human subjects was carried out in accordance with the Declaration of Helsinki (2008) of the World Medical Association.

***Generation and verification of liver-specific human IL32γ transgenic mice***

Because IL32γ is the most active isoform among the human IL32 variants that mediates the expression of cytokines [3-7], it was used to generate liver-specific human *IL32γ* transgenic (*hIL32γLTg*) mice (Shanghai Model Organisms Center, Inc., Shanghai, China) according to recent reports (*hIL32*-TG mice but not liver-specific) [7, Suppl. Ref. 2-3], as mice do not have an *IL32* orthologous gene. Briefly, the capped Piggybac transposase mRNA was in vitro transcribed using the mMESSAGE mMACHINE kit (Ambion, Austin, TX, USA) according to the manufacturer’s instructions. The transposases mRNA was microinjected into fertilized C57BL/6J oocytes with PB-LIVE-*IL32γ* vector (Suppl. Fig. 2A). The transgenic mice were identified by polymerase chain reaction (PCR) using the following primer pair: P1(5’-ATGTGCTTCCCGAAGGTCCTCTCT-3’)/P2(5’-TCATTTTGAGGATTGGGGTTAGA-3’) with a 705-bp PCR product (Suppl. Fig. 2B). Finally, the successful generation of *hIL32γLTg* mice was confirmed via western blotting analysis (Suppl. Fig. 2C).

All experiments involving animals were performed under the guidelines of the animal care and use committees at the Medical Research Center (Southwest Hospital, Chongqing, China). The study protocol with involvement of experimental animals was reviewed and approved by the Institutional Animal Care and Use Committee of the Southwest Hospital (Chongqing, China).

***Generation and verification of Abcb4 knockout mice***

*Abcb4* knockout mice (*Abcb4*-KO, C57BL/6J background) were developed by Shanghai Model Organisms Center, Inc (Shanghai, China). In brief, Cas9 mRNA was transcribed *in vitro* with the mMESSAGE mMACHINE T7 Ultra Kit (Ambion) according to the manufacturer’s instructions. The target sequences of two sgRNAs were 5'- CTACAAGGACATCTGGATACAGG-3' and 5'-GTTTGCCTAATTGTAAACGATGG-3'. The sgRNAs targeting exon3 of the *Abcb4* gene were designed using the online tool at<http://crispr.mit.edu/> (Suppl. Fig. 11A). The 411-bp deletion in exon3 resulted in a frameshift mutation of *Abcb4* and inactivated the *Abcb4* gene. The generation of homozygous *Abcb4*-KO mice was confirmed by PCR using the primer set P1 (5’-ATGGGTATTTGGGGAGCATTTT-3’)/P2(5’-CCCTTCCTGCATTTGCTTTCTA-3’) and yielded a 1177-bp PCR product (Suppl. Fig. 11B). Finally, *Abcb4*-KO mice were further validated by western blotting analysis (Suppl. Fig. 11C).

***Experimental animals***

Male C57BL/6J mice at age 8 weeks were purchased from the Center of Laboratory Animals of the Southwest Hospital (Chongqing, China). Upon arrival, mice were housed for 1 week prior to experiments, to allow them to adapt to their new environment. (1) For bile duct ligation (BDL) experiments, wild-type (WT) and *hIL32γLTg* mice were divided into four groups: sham operation or BDL for each mouse type (n=7–12 in each group, performed at 3 days post-operation). (2) For the 14-day 1% CA-feeding experiments, WT mice and *hIL32γLTg* mice were each divided into two groups: control diet (n=5-6, for each genotype) and 1% CA feeding (n=7 for WT group, n=9 for *hIL32γLTg* group). The mice were fasted overnight before they were sacrificed. Serum was collected and immediately stored at -80°C until analyses were performed. Biochemistry tests were carried out by the Department of Clinical Laboratory Medicine at the Southwest Hospital (Chongqing, China) [Suppl. Ref. 1]. The collected liver tissues were quickly perfused with phosphate-buffered saline (PBS) to flush out blood and then immediately cut into small pieces and rapidly frozen in liquid nitrogen until analyses were performed.

***Preparation and administration of AAV8-hIL32γ vector in Abcb4-KO mice***

AAV8*-hIL32γ* and AAV8*-CTR* were designed, produced, and purified by GENCHEM, Co., Ltd. (Shanghai, China). Briefly, a transgene expression cassette was assembled by cloning the *hIL32γ* cDNA (NCBI Gene ID:NM_001012635) into a modified AAV expression vector that includes the WT *AAV2* inverted terminal repeats (ITRs), a *CMV bGlobin* promoter, a *T2A* sequence, a luciferase gene, and a simian virus 40 polyadenylation signal (SV40polyA) (Suppl. Fig. 13A). *hIL32γ* cDNA was amplified using the following primers: *hIL32γ* forward primer5’-GAGGTAGTGGAATGGATCCCGCCACCATGTGCTTCCCGAAGGTCCTCTCTGATGAC-3’; *hIL32γ* reverse primer 5’-GCCTCAGCTATTTAAAGTTACACGGCGA TCTTTCCGCCCTTCTTGG-3’. Viral titers in terms of genome copies per milliliter were determined by quantitative (q)PCR using the following primers: *CMV* forward primer 5'-TATTAGTCATCGCTATTACC-3'; *CMV* reverse primer 5'-TGAGTCAAACCGCTATCC -3'; *Luciferase* forward primer 5’-CTGGGACGAAGACGAACAC-3’; and *Luciferase* reverse primer 5’- GAAGACCTGCGACACCTG-3’.

For AAV8-*hIL32γ* gene therapy (Suppl. Fig. 13B), 8-week-old *Abcb4*-KO mice received intravenous administration of AAV8*-hIL32γ* (n=7, 4 male and 3 female) or AAV8*-CTR* (n=6, 3 male and 3 female) at a dose of 0.685×10^13^ vg/kg. Meanwhile, WT mice were intravenously injected with saline (n=7, 4 male and 3 female) as negative control group. Two weeks after AAV8 vector injection, all animals were intraperitoneally injected with D-Luciferine at 150 mg/kg dissolved in saline (Shanghai Sciencelight Biology Science&Technology Co.,Ltd., China) and then the high expression of liver IL32 was verified by bioluminescence measurement (Suppl. Fig. 14A). Four weeks following AAV8 injection, all the studied mice were sacrificed, and hepatic IL32 expression was determined by TaqMan qPCR analysis to confirm overexpression of hepatic IL32 (Suppl. Fig. 14B).

***Preparation and treatment of primary mouse hepatocytes***

Primary mouse hepatocytes were isolated from 10- and 20-week-old mice (WT and *hIL32γLTg*) using collagenase perfusion as previously described [10]. Isolated hepatocytes were cultured in 5% fetal bovine serum (FBS)-Williams’ Medium E. After pretreatment with or without 5μM Anisomycin, an agonist of JNK/MAPK signaling, for 30min, these cells were treated with 25μM conjugate BAs, including taurocholic acid (TCA), glycocholate acid (GCA), taurochenodeoxycholate acid (TCDCA), and glycochenodexycholate acid (GCDCA) (Sigma-Aldrich Chemical Co., St. Louis, MO, USA) for 12 or 24 h. Finally, the culture supernatant, total RNA, and whole cell lysates were collected as described previously [10, Suppl. Ref. 1] for use in enzyme-linked immunosorbent assays (ELISAs), TaqMan qPCR and western blotting analysis, respectively.

***Flow cytometric analysis***

The liver tissues of WT and TG mice were perfused with Hank’s Balanced Salt Solution (HBSS) via the portal vein for 10 min, followed by digestion using collagenase type II (Worthington Biochemical Corporation, Freehold, NJ, USA) perfusion as described previously [10]. Isolated cells were centrifuged at 40*g* for 30 min to pellet the parenchymal fraction. The single-cell suspensions were isolated by Percoll gradient in order to collect the nonparenchymal (NPC) fraction. The obtained cells were incubated with the antibodies against CD45-APC-Cy7 (clone 30-F11), Ly-6G/Ly-6C (Gr-1)-FITC (clone RB6-8C5), CD11b-PE-Cy7 (clone M1/70), F4/80-PE (clone T45-2342), CD3e-PerCP-Cy5.5 (clone 145-2C11), NK1.1-APC (clone PK136), and/or CD45R/B220-PE (clone RA3-6B2) antibodies (all from BD Biosciences, San Jose, CA, USA) in fluorescence-activated cell sorting (FACS) buffer for 30 min at 4°C in the dark. Stained cells were analyzed on a BD FACS Aria II flow cytometer (BD Biosciences, San Jose, CA). The CD45+ cells were first gated from the NPC single-cell suspension, followed by use of CD3+ gating for T lymphocytes, B220+ gating for B lymphocytes, NK1.1+ gating for natural killer NK cells, F4/80+ CD11b+ gating for macrophages, and Gr-1+ CD11b+ gating for neutrophils. Data were analyzed using FlowJo software 10.03 (Tree Star Inc., Ashland, OR, USA).

***Examination of Ccl2, Cxcl5, and Cxcl10 levels in the culture supernatant of primary mouse hepatocytes treated with conjugate BAs***

Primary mouse hepatocytes were isolated from WT and TG mice and treated with 25μM TCA, GCA, TCDCA, or GCDCA for 24 h. After treatment, the culture supernatant was collected and cytokine expression levels were determined using a Ccl2 enzyme-linked immunosorbent assay (ELISA) Kit (Proteintech Group, Inc., Chicago, IL) and Cxcl5 and Cxcl10 ELISA Kit (Cloud-Clone Corp., Wuhan, China) according to the manufacturer’s instructions.

***Bioluminescence measurement in living mice***

Bioluminescence measurement was performed as previously described [Suppl. Ref. 4]. In brief, mice were given an intraperitoneal injection of 150 mg/kg D-Luciferine (Catalog#luc001, Shanghai Sciencelight Biology Science&Technology Co., Ltd., China), dissolved in saline and anesthetized with 2.5% isoflurane (RWD Life Science Co., Ltd., Shenzhen, China) vaporized in constant O_2_ with a flow rate of 0.5 L/min. Ten minutes after injection, all the experimental mice were placed facing up (belly side up) in an IVIS Spectrum imaging system (Caliper Life Sciences, Hopkinton, MA, USA), and luciferase activity was determined using a highly sensitive charged coupled device (CCD) camera with an exposure time of 0.2 s. Images were processed using Living Image software 4.4 (Caliper Life Sciences). Light intensity was quantified as photons/s/sr/cm^2^.

***RNA extraction, reverse transcription, and quantitative real-time polymerase chain reaction***

Total RNA was extracted from liver tissues or primary mouse hepatocytes using Trizol reagent (Invitrogen, Carlsbad, CA, USA) according to the manufacturer’s instructions. The cDNA was synthesized and analyzed by real-time qPCR as described previously [Suppl. Ref. 1]. The TaqMan probes (Life Technologies Co., Carlsbad, CA, USA) and SYBR primers used in this study are listed in Table.S4. Glyceraldehyde-3-phosphate dehydrogenase (GAPDH) was used as the reference gene for normalizing the gene expression.

***Western blot analysis***

Liver tissues and whole cell lysates were prepared as described previously [Suppl. Ref. 1, Suppl. Ref. 5]. Nuclear extractions were obtained using commercial kits from Thermo Scientific (Waltham, MA, USA) according to the manufacturer’s instructions [Suppl. Ref. 1, Suppl. Ref. 5]. All protein samples were separated by sodium dodecyl sulfate-polyacrylamide gel electrophoresis (SDS-PAGE) and transferred to polyvinylidene difluoride (PVDF) membranes (0.22 μm). The sources of primary antibodies and the dilutions used in this study are listed in Table.S5.

***Chromatin immunoprecipitation assays***

Chromatin immunoprecipitation (ChIP) assays were performed using a ChIP Assay Kit (Millipore, Bedford, MA, USA) according to the manufacturer’s instructions. Soluble chromatins were prepared from human or mouse liver tissues. The chromatins were immunoprecipitated using antibodies against FXR and c-Jun (Table.S5). The primers and sizes of the amplification products are listed in Table.S6. These assays were performed as previously described [Suppl. Ref. 1, Suppl. Ref. 6].

***Co-immunoprecipitation assays***

The liver tissues were homogenized in radio-immunoprecipitation assay (RIPA) buffer (Sigma-Aldrich) containing Complete EDTA-free Protease and PhosSTOP Phosphatase inhibitors (Roche, Palo Alto, CA, USA). The homogenates were subjected to co-immunoprecipitation (Co-IP) using anti-FXR antibody (Table.S5), as described previously [Suppl. Ref. 5].

***Immunohistochemistry analysis***

Immunohistochemistry (IHC) analysis was performed as previously described [Suppl. Ref. 1, Suppl. Ref. 5]. The dilutions of the primary antibodies used in this study are presented in Table.S5.

***LC-MS/MS analysis of 7-alpha-C4 in mouse liver tissues extracts***

Levels of 7-alpha-C4 (7alpha-hydroxy-4-cholesten-3-one, 7-alpha-C4) in mouse liver were determined by the Shanghai Omicspro Biotech Company (Shanghai, China) as described previously [Suppl. Ref. 1, Suppl. Ref. 7].

***Sirius Red, Masson Trichrome and H&E staining for mouse liver histology analysis***

Sirius Red, Masson Trichrome, and H&E staining in mouse liver samples were performed as described previously [Suppl. Ref. 1, Suppl. Ref. 8].

***Statistical analysis***

All data were analyzed using the independent-samples Student’s *t*-test (two-tailed) and expressed as mean ± standard deviation (SD). Linear regression analysis was also performed using SPSS software (PASW Statistics 18, IBM; SPSS, Inc., Chicago, IL). A value of *p*<0.05 was considered statistically significant.

**Suppl. References**

1. **Pan Q, Zhang XX, Zhang LJ**, et al. Solute carrier organic anion transporter family member 3A1 is a bile acid efflux transporter in cholestasis. *Gastroenterology*. 2018;155:1578–1592.

2. Lee DH, Hong JE, Yun HM, et al. Interleukin-32β ameliorates metabolic disorder and liver damage in mice fed high-fat diet. *Obesity (Silver Spring).* 2015;23(3):615-622.

3. Lee DH, Kim DH, Hwang CJ, et al. Interleukin-32γ attenuates ethanol-induced liver injury by the inhibition of cytochrome P450 2E1 expression and inflammatory responses. *Clin Sci (Lond)*. 2015;128(10):695-706.

4. Murillo O, Luqui DM, Gazquez C, et al. Long-term metabolic correction of Wilson’s disease in a murine model by gene therapy. *J Hepatol*. 2016;64(2):419-426.

5. Chai J, Cai SY, Liu XC, et al. Canalicular membrane MRP2/ABCC2 internalization is determined by Ezrin Thr567 phosphorylation in human obstructive cholestasis. *J Hepatol*. 2015; 63(6):1440–1448.

6. Pan Q, Tian Y, Li X, et al. Enhanced membrane-tethered mucin 3 (MUC3) expression by a tetrameric branched peptide with a conserved TFLK motif inhibits bacteria adherence. *J Biol Chem*. 2013; 288(8):5407-5416.

7. Camilleri M, Nadeau A, Tremaine WJ, et al. Measurement of serum 7alpha-hydroxy-4-cholesten-3-one (or 7alphaC4), a surrogate test for bile acid malabsorption in health, ileal disease and irritable bowel syndrome using liquid chromatography-tandem mass spectrometry. *Neurogastroenterol Motil*. 2009, 21(7):734-e43.

8. Zhang GZ, Sun HC, Zheng LB, et al. In vivo hepatic differentiation potential of human umbilical cord-derived mesenchymal stem cells: Therapeutic effect on liver fibrosis/cirrhosis. *World J Gastroenterol*. 2017; 23(46):8152-8168.

**Suppl. Figures and Figure legends:**


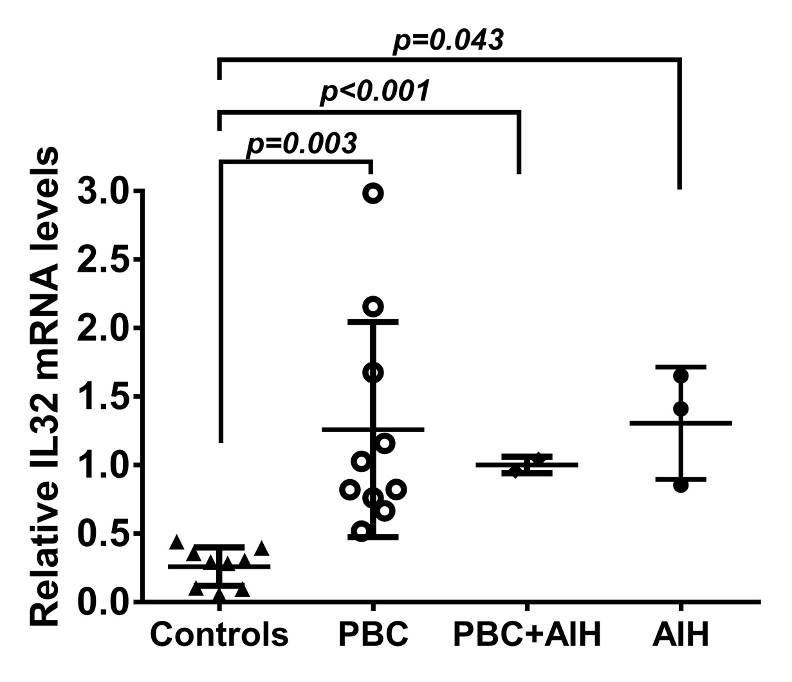


**Suppl. Figure 1.** Hepatic IL32 mRNA levels were increased in patients with PBC (n=10), AIH (n=3) and PBC plus AIH (n=2), when compared to controls.


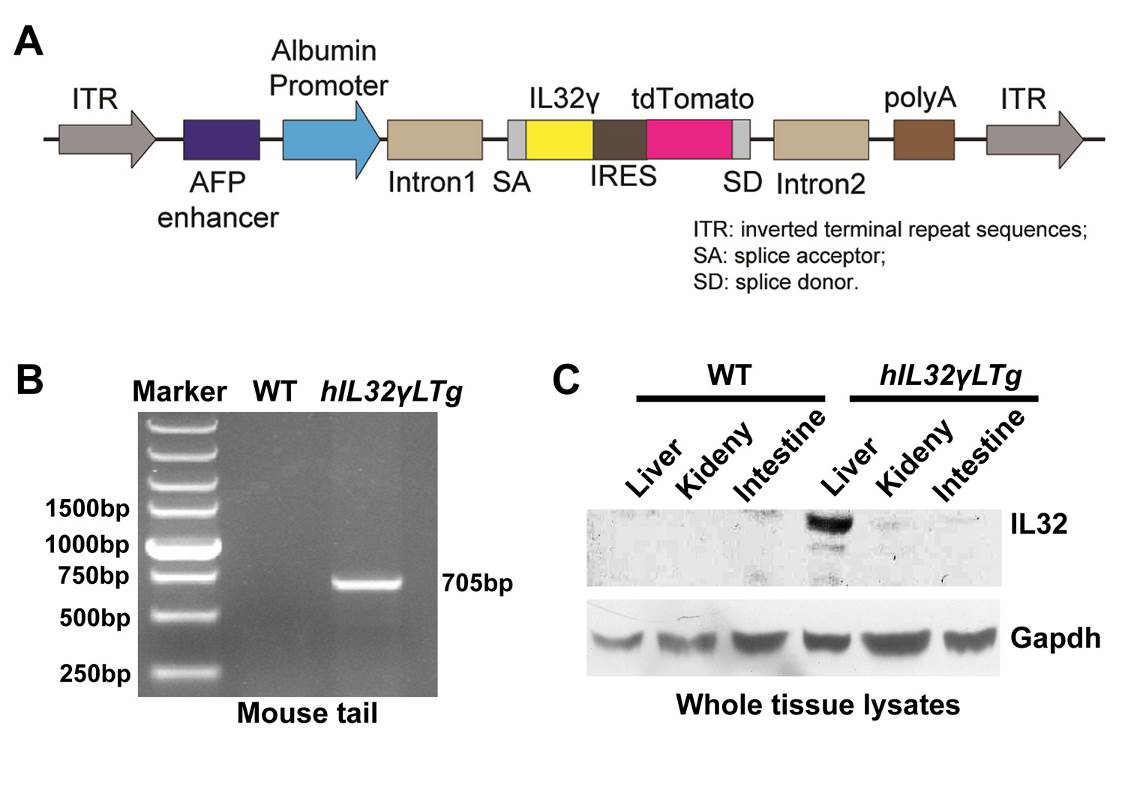


**Suppl. Figure 2.** Generation and validation of liver-specific human *IL32γ* transgenic (*hIL32γLTg*) mice. (A) Schematic diagram of the expression vector used for generation of *hIL32γLTg* mice. (B) Genotyping of *hIL32γLTg* mice. PCR was performed using genomic DNA extracted from mouse tail biopsies. (C) Western blot analysis confirmed the overexpression of hepatic IL32 protein in *hIL32γLTg* mice.

**Suppl. Figure 3.** IL32 overexpression induced the expression of nuclear receptor Fxr target genes Shp and Ostβ, without altering the mRNA levels of Fxr in *hIL32γLTg* compared to WT primary mouse hepatocytes treated with 25μM conjugate BAs (TCA) (B). ******p<*0.05 vs. WT-DMSO; **#***p<*0.05 vs. WT-TCA. WT, wild-type primary mouse hepatocytes; TG, *hIL32γLTg* primary mouse hepatocytes.


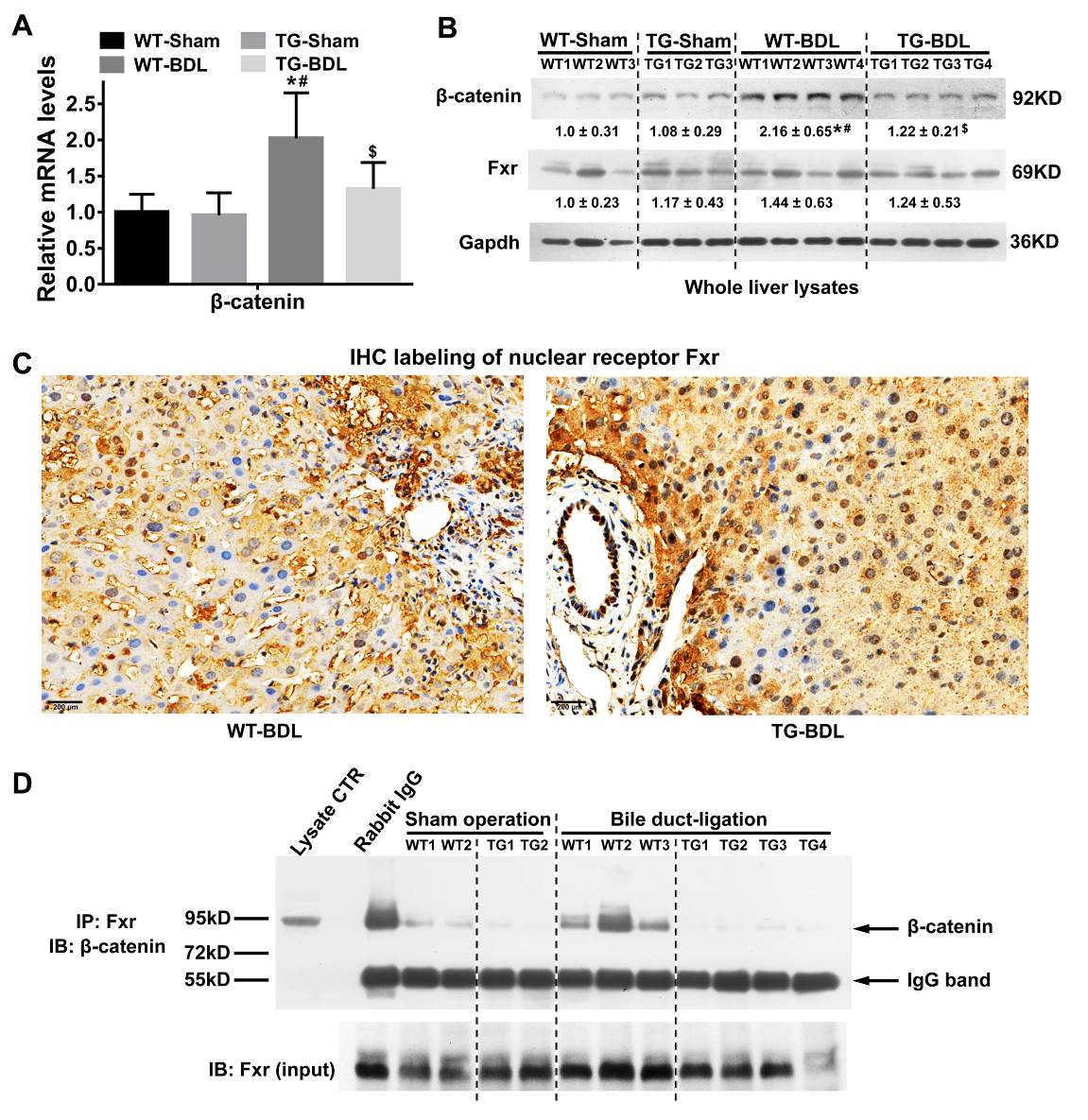


**Suppl. Figure 4.** Overexpression of hepatic IL32 in BDL mice mediated Fxr nuclear translocation by enhancing Fxr/β-catenin protein complex dissociation. (A) Hepatic mRNA levels of β-catenin and (B) protein levels of β-catenin and Fxr were determined in Sham and BDL mice. Sham operation WT group (n=7); TG-Sham, sham operation *hIL32γLTg* group (n=8); WT-BDL, bile duct-ligated wild-type group (n=12); TG-BDL, bile duct-ligated *hIL32γLTg* group (n=11). ******p<*0.05 vs. WT-Sham group; **#***p<*0.05 vs. TG-Sham group; **$***p<*0.05 vs. WT-BDL group. (C) IHC labeling of Fxr protein in the liver of a WT-BDL mouse (left) and a TG-BDL mouse (right). Increased expression of Fxr in the nuclei was observed in the liver of TG-BDL mice compared with TG-WT mice. WT-BDL, bile duct-ligated wild-type mice; TG-BDL, bile duct-ligated *hIL32γLTg* mice. (D) Overexpression of hepatic IL32 enhanced the dissociation of the Fxr/β-catenin protein complexes, as β-catenin was lower in the Fxr precipitated complexes from TG mice compared with WT mice after BDL. IP, immunoprecipitation; IB, immunoblot; Lysate CTR, whole liver tissue lysates as a positive control; Rabbit IgG, normal IgG as a negative control; WT, wild-type mice; TG, *hIL32γLTg* mice.


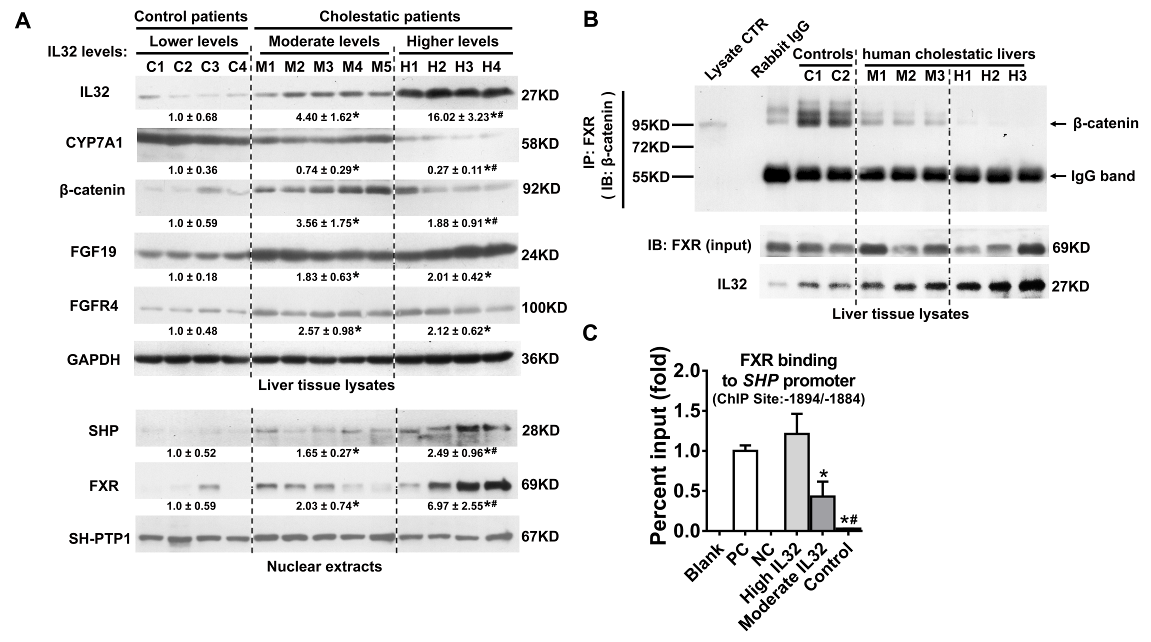


**Suppl. Figure 5.** Hepatic BA synthesis was repressed in obstructive cholestatic patients with elevated levels of hepatic IL32. (A) Representative western blots and corresponding densitometry of liver IL32, CYP7A1, β-catenin, FGF19, and FGFR4 from liver tissue lysates and nuclear receptors SHP and FXR from liver tissue nuclear extracts. *C1-4,* control livers with low levels of IL32; *M1-5,* obstructive cholestasis liver tissues with moderately elevated levels of IL32; *H1-4,* obstructive cholestasis liver tissues with higher levels of IL32. ******p<*0.05 vs. control groups; **#***p<*0.05 vs. moderate levels of hepatic IL32. (B) FXR/β-catenin protein complex dissociation in human cholestatic liver tissues was gradually increased along with increasing IL32 expression. IP, immunoprecipitation; IB, immunoblot; Lysate CTR, Whole liver tissue lysates as a positive control; Rabbit IgG, Normal IgG as a negative control. *C1-2,* control liver tissues with low levels of IL32; *M1-3,* obstructive cholestasis liver tissues with moderate levels of IL32; *H1-3,* obstructive cholestasis liver tissues with higher levels of IL32. (C) The binding activity of FXR to the *SHP* promoter in human cholestatic liver tissues was gradually increased along with the elevated amount of IL32 expression. ******p<*0.05 vs. higher levels of hepatic IL32; **#***p<*0.05 vs. moderate levels of hepatic IL32.


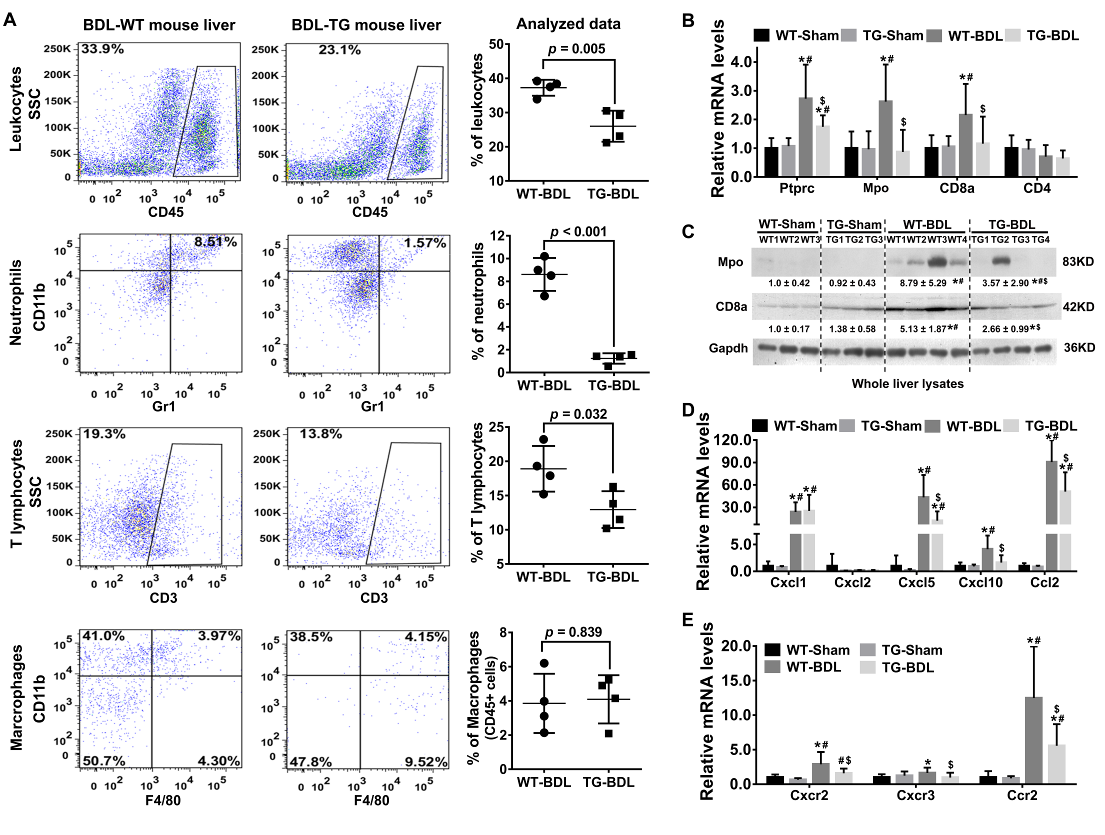


**Suppl. Figure 6.** Hepatic IL32 overexpression attenuated the cholestatic liver inflammatory response. (A) Representative flow cytometry plots (left) and their corresponding analysis (right) for total leukocytes (CD45+ cells), neutrophils (CD11b+ and Gr1+ cells), T lymphocytes (CD3+ cells), and macrophages (CD11b+ and F4/80+ cells) in the liver tissues of WT and *hIL32γLTg* mice after BDL (n=4 per group). The numbers of total leukocytes, neutrophils, and T lymphocytes in the livers of TG-BDL mice were significantly lower than those in BDL-WT mice. However, there were no differences in the numbers of B lymphocytes (B200+ cells) and natural killer (NK) cells (NK1.1+ cells) between these two groups (Suppl. Fig. 7A&B). (B) Hepatic mRNA and (C) protein levels of Ptprc (total leukocyte marker), MPO (neutrophil marker), CD8a (CD8+ T-cell marker), and/or CD4 (CD4+ T-cell marker). (D) Liver mRNA levels of chemokines Cxcl1, Cxcl2, Cxcl5, Cxcl10, and Ccl2, and (E) their receptors Cxcr2, Cxcr3, and Ccr2. WT-Sham, Sham operation WT group (n=7); TG-Sham, sham operation *hIL32γLTg* group (n=8); WT-BDL, bile duct-ligated wild-type group (n=12); TG-BDL, bile duct-ligated *hIL32γLTg* group (n=11). ******p<*0.05 vs. WT-Sham group; **#***p<*0.05 vs.TG-Sham group; **$***p<*0.05 vs. WT-BDL group.


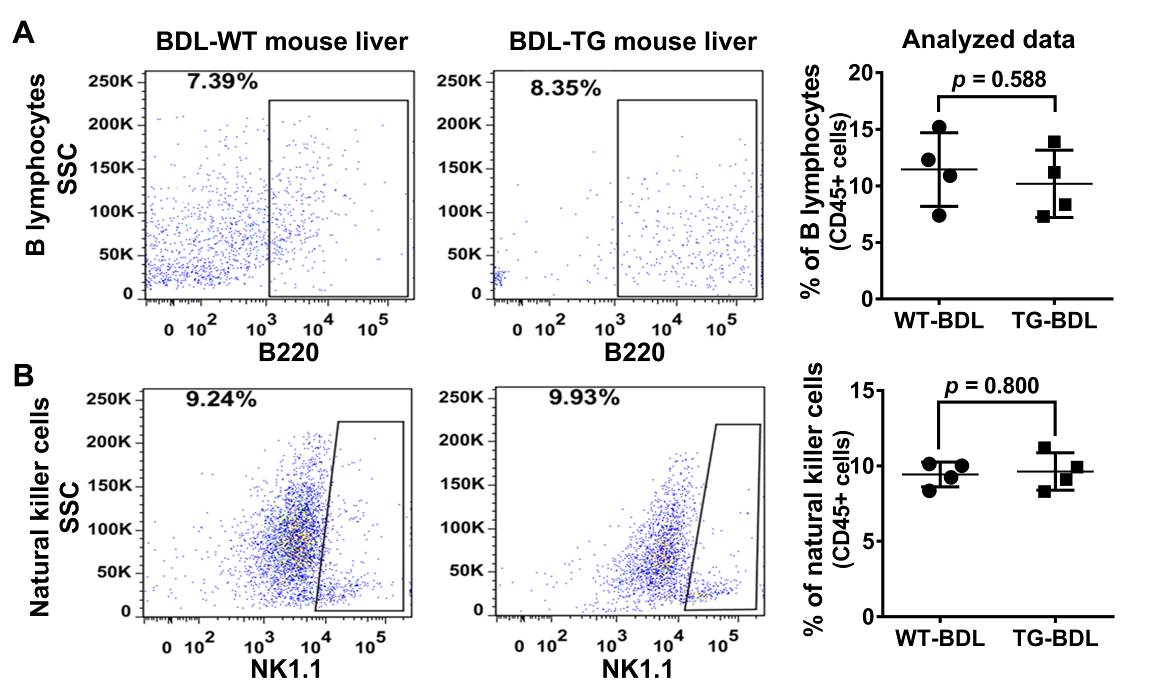


**Suppl. Figure 7.** Overexpression of hepatic IL32 reduced liver inflammation in mice after bile duct-ligation. (A&B) Representative flow cytometry plots (left) and their corresponding analyses (right) for B lymphocytes (B200+ cells) and NK cells (NK1.1+ cells) in WT and TG mouse livers at 3 days after BDL (n=4 per group). To avoid the interference from blood cells, cholestatic mouse liver tissues were isolated by collagenase type II solution after perfusion with Hank’s balanced salt solution. Isolated mouse nonparenchymal cells were immediately used for flow cytometric analysis.


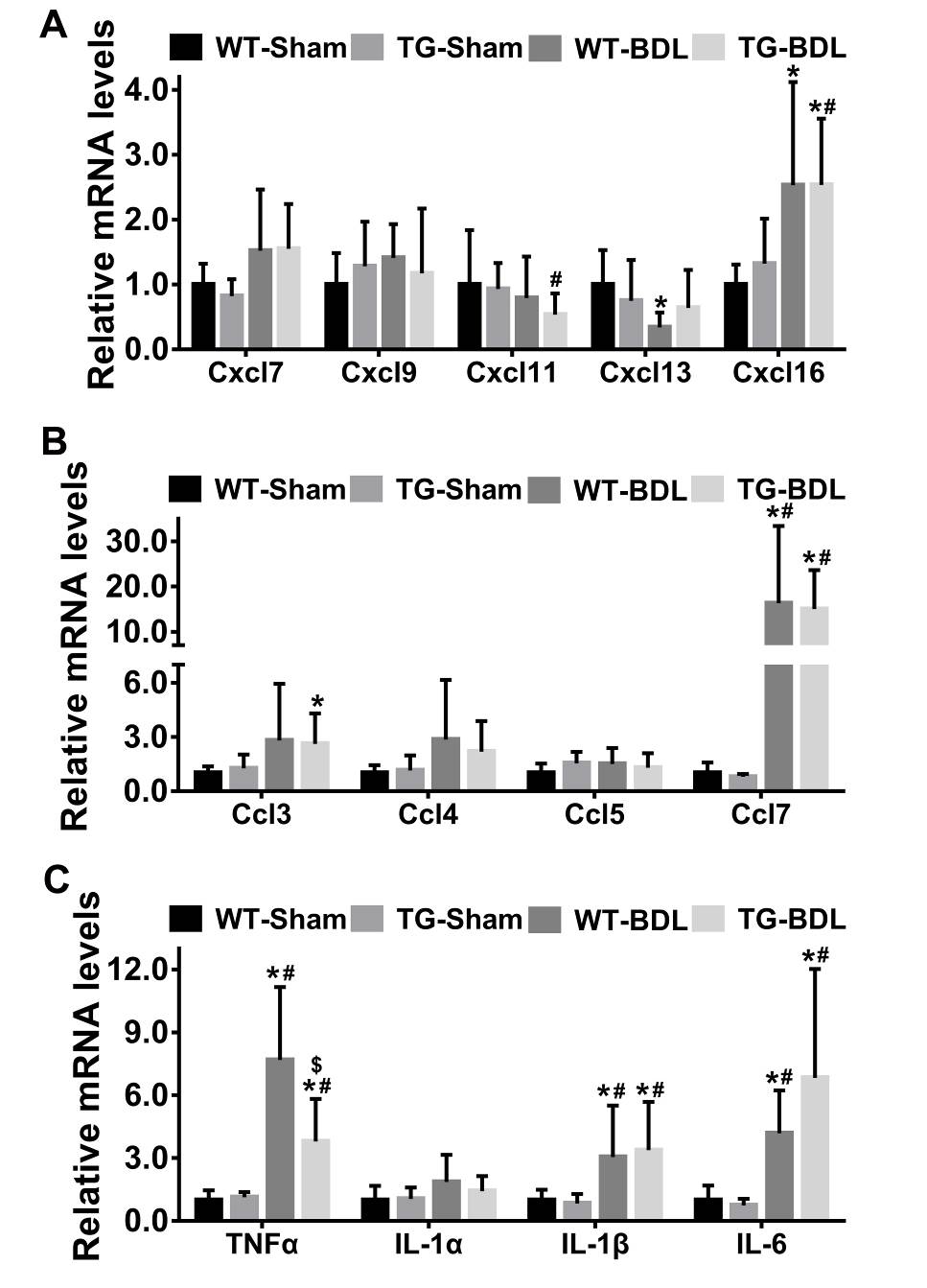


**Suppl. Figure 8.** Hepatic mRNA levels of the Cxcl family members Cxcl7, Cxcl9, Cxcl11, Cxcl13, and Cxcl16; (B) chemokine Ccl family members Ccl3, Ccl4, Ccl5, and Ccl7; and (C) inflammatory cytokines TNFα, IL-1α, IL-1β, and IL-6 were determined. Sham operation WT group (n=7); TG-Sham, sham operation *hIL32γLTg* group (n=8); WT-BDL, bile duct-ligated wild-type group (n=12); TG-BDL, bile duct-ligated *hIL32γLTg* group (n=11). ******p<*0.05 vs. WT-Sham group; **#***p<*0.05 vs. TG-Sham group; **$***p<*0.05 vs. WT-BDL group.


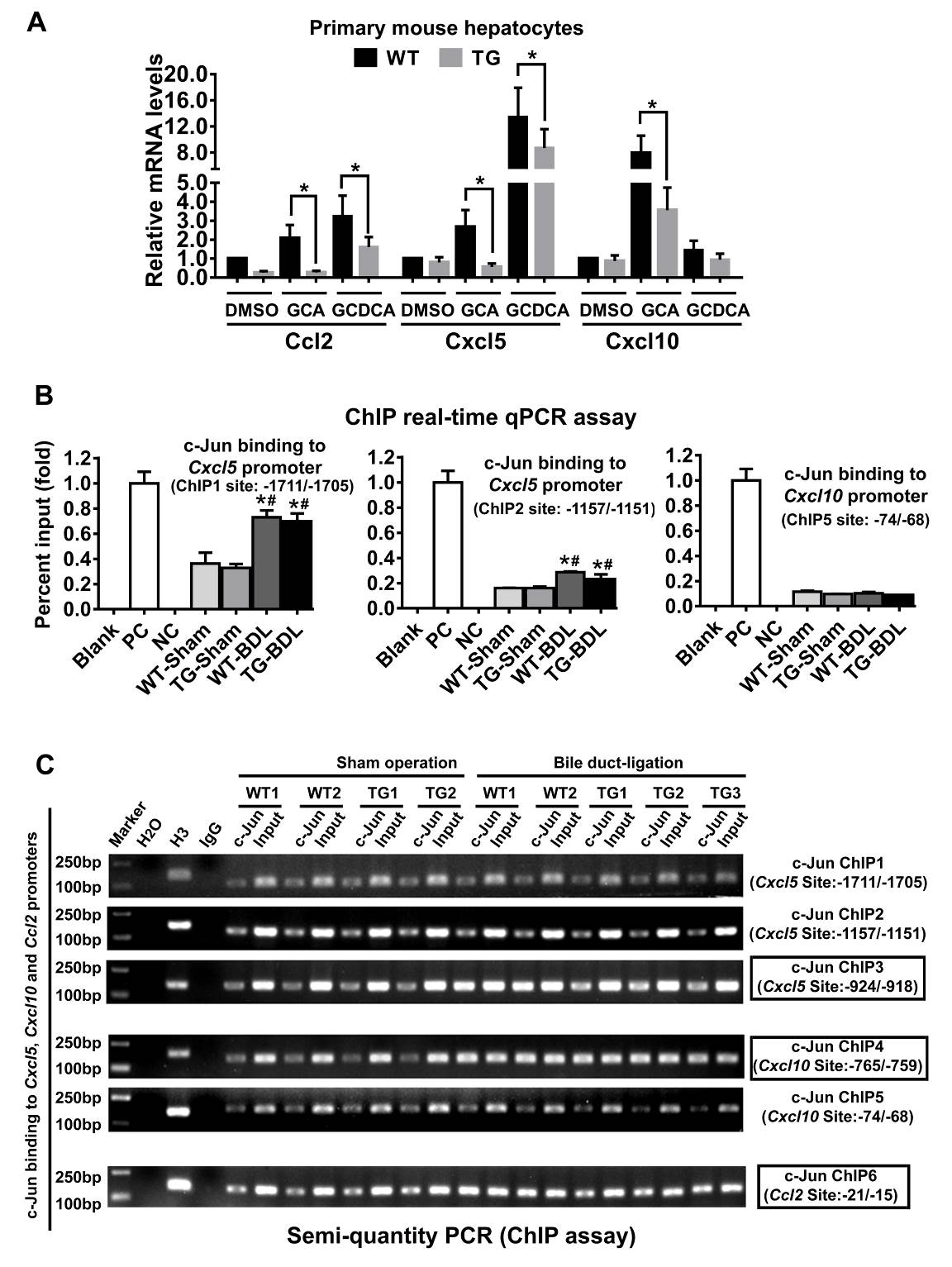


**Suppl. Figure 9.** (A) mRNA levels of chemokines Cxcl5, Cxcl10, and Ccl2 in WT and *hIL32γLTg* primary mouse hepatocytes treated with 25μM conjugate BAs GCA and GCDCA for 12 h. **p<0.05*, n=3. (B) ChIP assay (real-time qPCR) results revealed that binding activity of c-Jun to the *Cxcl10* (ChIP5 site -74/-68) or *Cxcl5* (ChIP1 site -1711/-1705 and ChIP2 site -1157/-1151) promoter did not differ between TG-BDL and WT-BDL mouse liver tissues. WT-Sham, sham operation WT group; TG-Sham, sham operation *hIL32γLTg* group; WT-BDL, bile duct-ligated wild-type group; TG-BDL, bile duct-ligated *hIL32γLTg* group. ******p<*0.05 vs. WT-Sham group; **#***p<*0.05 vs. TG-Sham group. (C) ChIP (real-time qPCR) assay results (Fig.3F and Suppl.Fig.9B) were verified by ChIP (semi-quantitative PCR) assay.


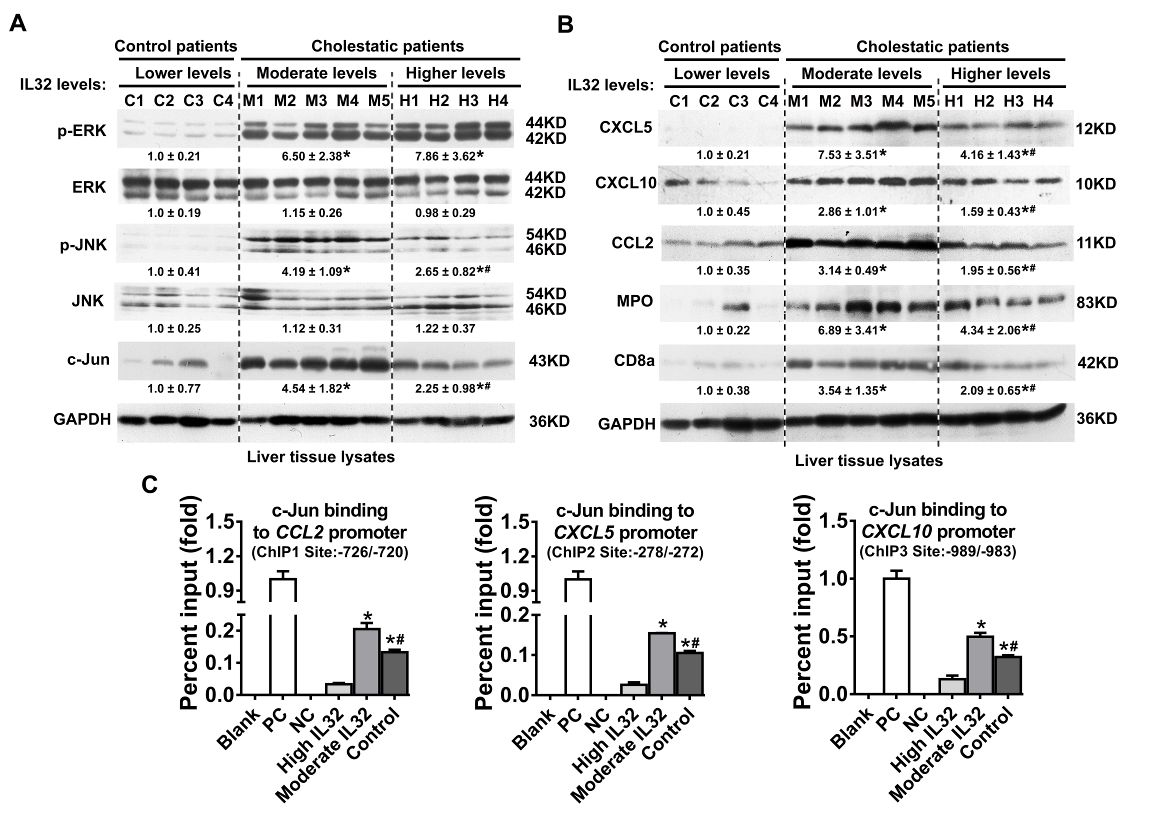


**Suppl. Figure 10.** A higher level of hepatic IL32 was significantly associated with lower hepatic inflammation in obstructive cholestatic patients. (A) Representative western blots and corresponding densitometry of phospho-ERK, ERK, phospho-JNK, JNK and c-Jun from human liver tissue lysates. (B) Representative western blots and corresponding densitometry of chemokines CXCL5, CXCL10, CCL2, neutrophil marker MPO, and CD8+ T-cell marker CD8a from human liver tissue lysates. *C1-4,* control livers with low levels of IL32; *M1-5,* obstructive cholestasis liver tissues with moderately elevated levels of IL32; *H1-4,* obstructive cholestasis liver tissues with higher levels of IL32. ******p<*0.05 vs. control groups; **#***p<*0.05 vs. moderate levels of hepatic IL32. (C) ChIP assays revealed that the binding activities of c-Jun to the *CCL2* (ChIP1 site -726/-720), *CXCL5* (ChIP2 site -278/-272)*,* and *CXCL10* (ChIP3 site -989/-983) promoters were lower in human cholestatic liver tissues with high IL32 expression than in those with moderate IL32 expression. ******p<*0.05 vs. higher levels of hepatic IL32; **#***p<*0.05 vs. moderate levels of hepatic IL32.


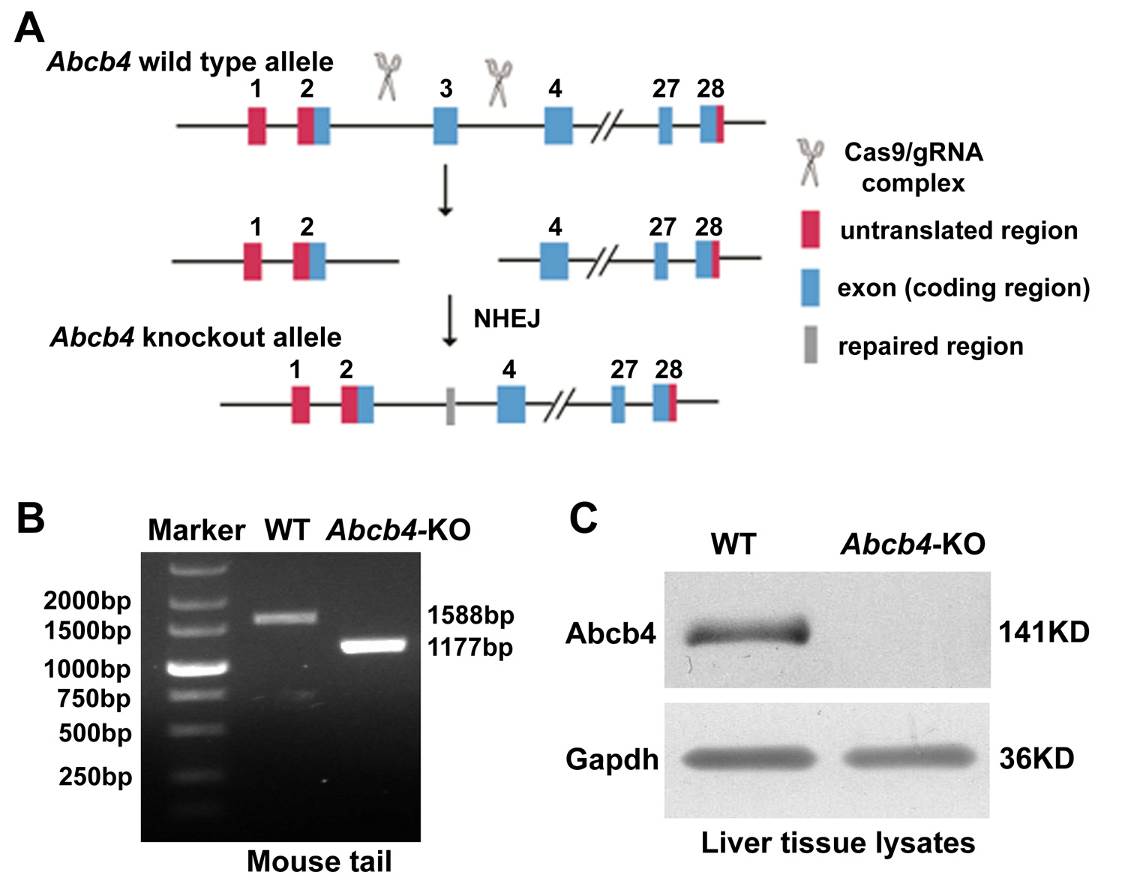


**Suppl. Figure 11.** Generation and validation of *Abcb4* knockout (KO) mice. (A) Schematic diagram of the expression vector used for generation of *Abcb4-*KO mice. (B) Genotyping of *Abcb4-*KO mice. PCR was performed using genomic DNA extracted from the mouse tail biopsies. (C) Western blot analysis confirmed the ablation of the Abcb4 protein in *Abcb4-*KO mice.


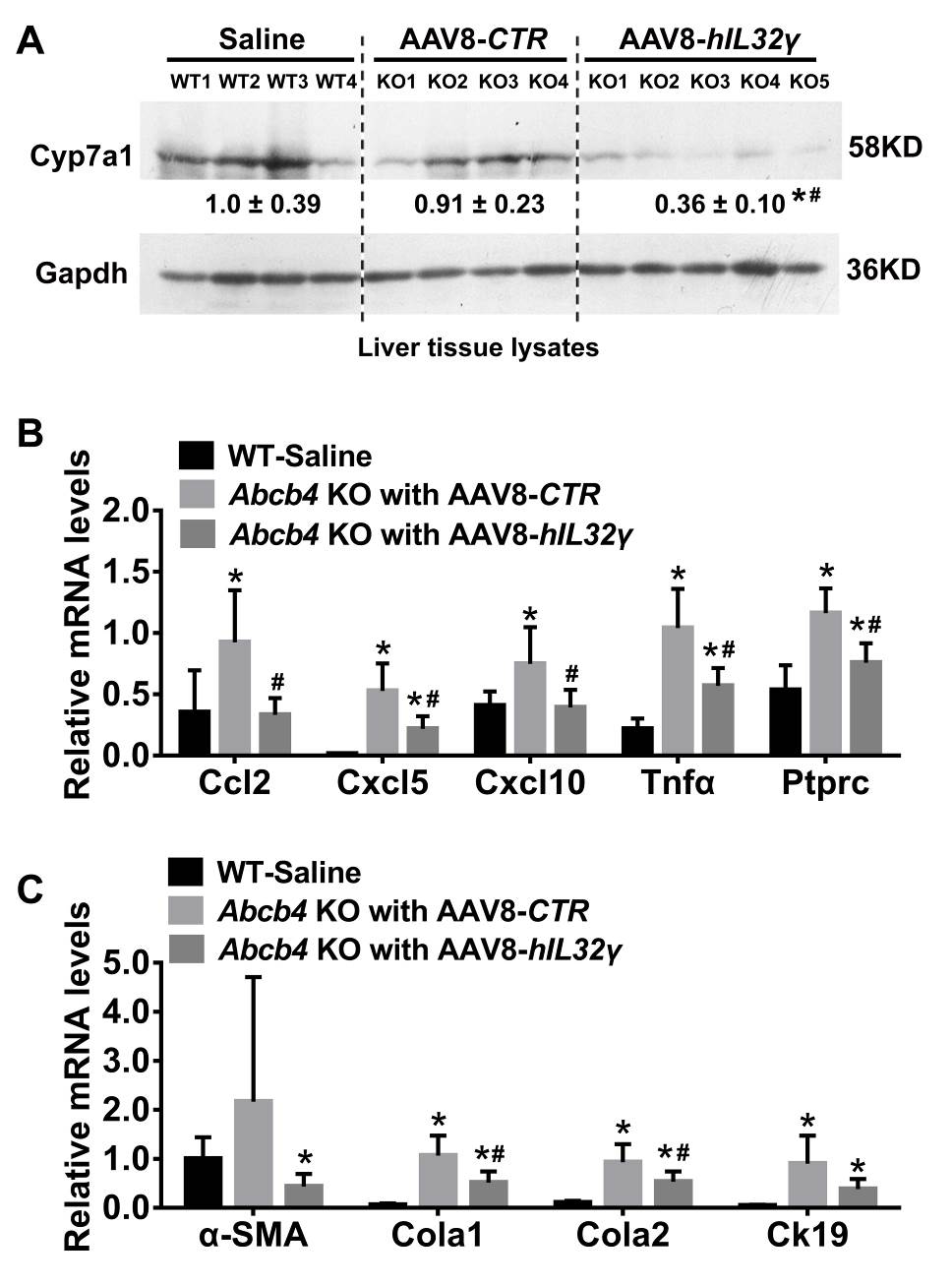


**Suppl. Figure 12.** AAV8-*hIL32γ* vector injection repressed BA synthesis and ameliorated cholestatic liver inflammation and fibrosis in *Abcb4* knockout mice. (A) Representative western blots for protein expression of the rate-limiting BA synthetic enzyme Cyp7a1; (B) hepatic mRNA levels of chemokines Ccl2, Cxcl5, Cxcl10, inflammatory cytokine TNFα, and total leukocytes marker Ptprc; and (C) liver mRNA levels of fibrotic genes α-SMA, Cola1, Cola2, and bile duct proliferation marker Ck19. WT, wild-type mice; KO, knockout mice. ******p<*0.05 vs. WT-Saline group; **#***p<*0.05 vs. *Abcb4*-KO with AAV8*-CTR* vector injection.


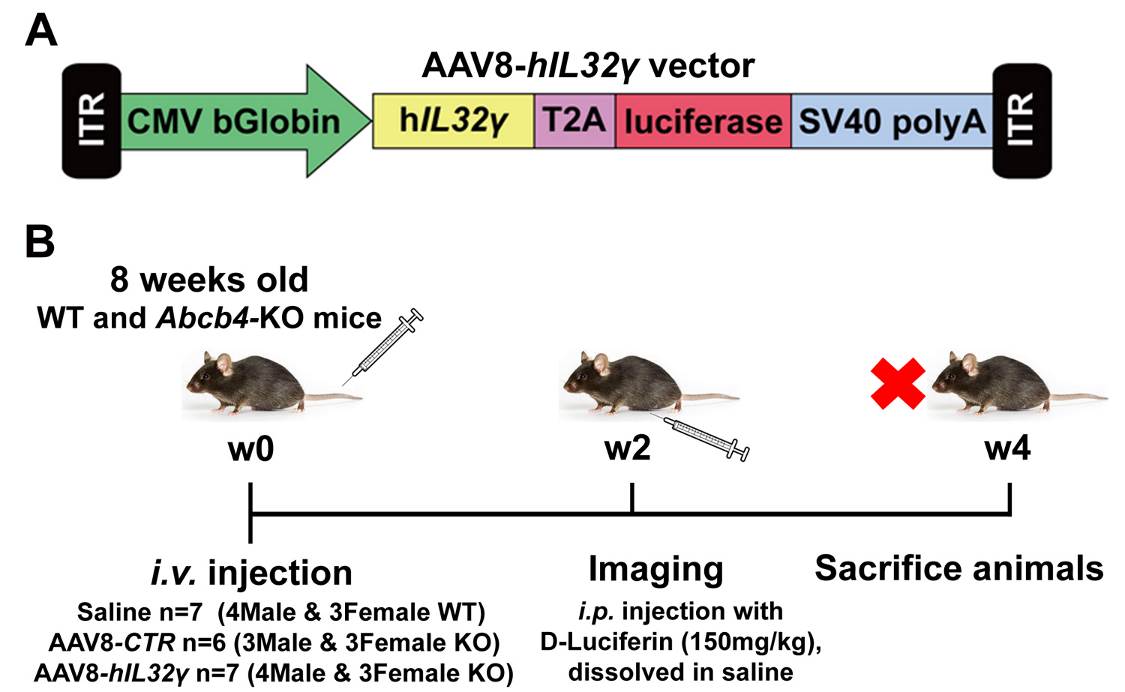


**Suppl. Figure 13.** Schematic diagrams of the expression vector and study design. (A) Schematic diagram of the AAV8-*hIL32γ* vector consisting of an expression cassette including a *CMV bGlobin* promoter, the *hIL32γ* coding sequence, a T2A sequence, a luciferase gene, and a simian virus 40 polyadenylation signal (SV40polyA), flanked by AAV2 ITRs. (B) At t=0, 8-week-old *Abcb4-*KO mice received intravenous administration of AAV8*- IL32γ* (n=7, 4 male and 3 female) or AAV8*-CTR* (n=6, 3 male and 3 female) at a dose of 0.685×10^13^ vg/kg. WT mice given intravenous saline (n=7, 4 male and 3 female) were used as the negative control. At t=2, all mice received an intraperitoneal injection of D-Luciferine at 150mg/kg (dissolved in saline), and then bioluminescence measurements were performed after 10 min. At t=4, all the studied mice were sacrificed and samples were collected for subsequent analysis.


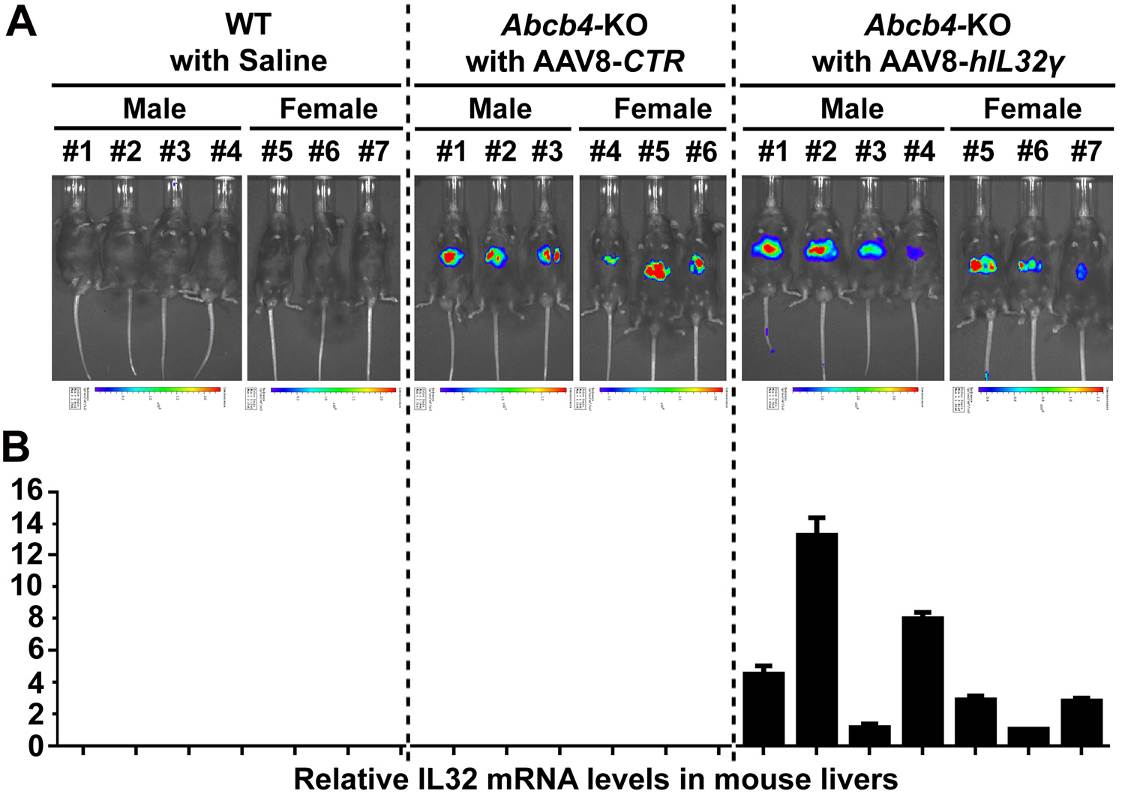


**Suppl. Figure 14.** Overexpression of hepatic IL32 in *Abcb4*-KO mice following intravenous injection of AAV8*-hIL32γ* vectors. (A) *Abcb4*-KO mice, a murine model of primary sclerosing cholangitis (PSC), were divided into two groups given: AAV8*-CTR* vector injection (n=6, 3 male and 3 female) or with AAV8*-hIL32γ* vector injection (n=7, 4 male and 3 female). WT mice given saline injection were used as a negative control group (n=7, 4 male and 3 female). (B) Hepatic mRNA levels of IL32 were measured by TaqMan qPCR. Hepatic overexpression of IL32 was determined in *Abcb4*-KO mice after AAV8*-hIL32γ* vector injection.


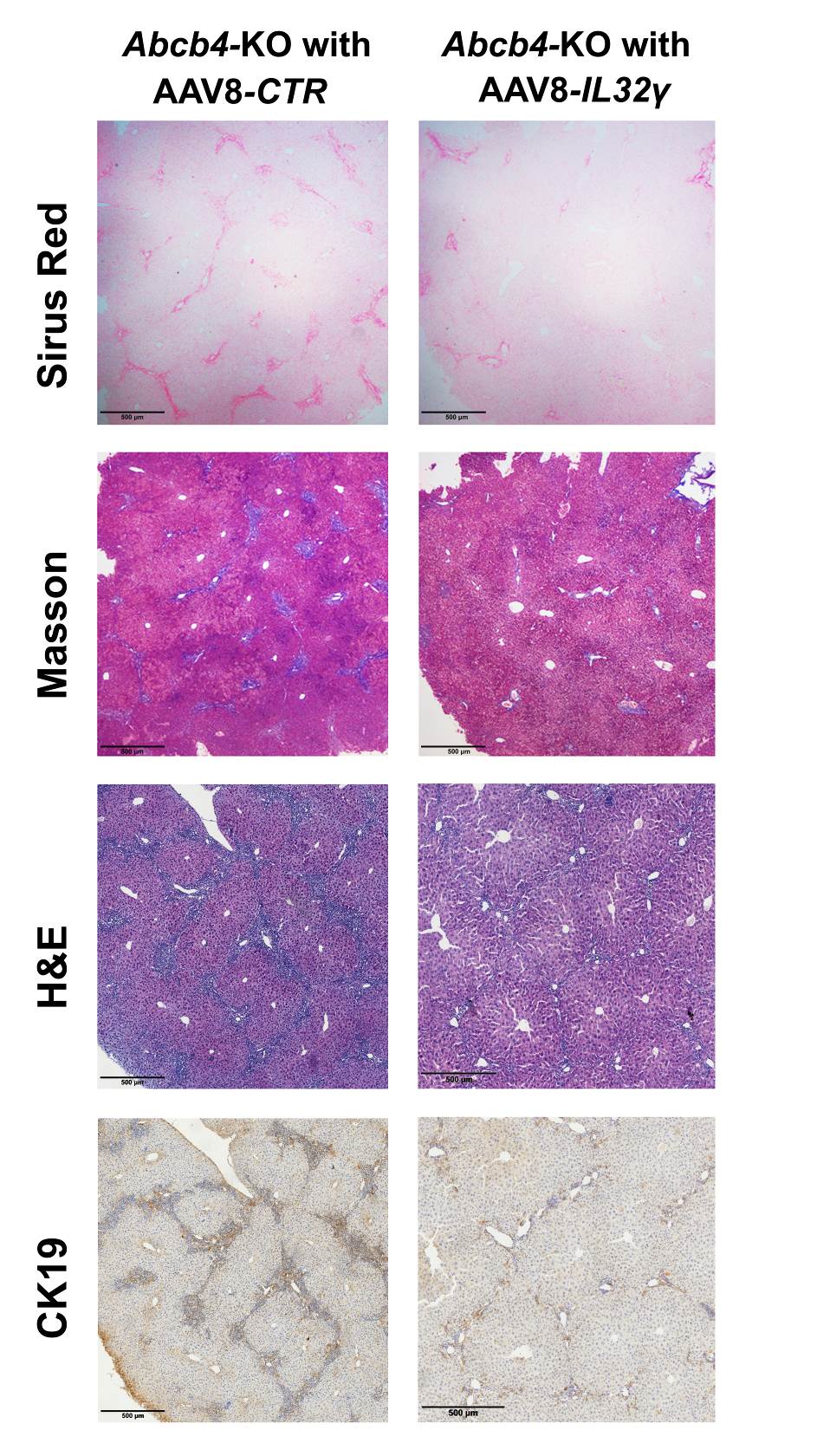


**Suppl. Figure 15.** Sirius Red, Masson Trichrome, and H&E staining of mouse liver tissues and IHC labeling of CK19 in mouse liver tissues. WT, wild-type mice; KO, knockout mice.


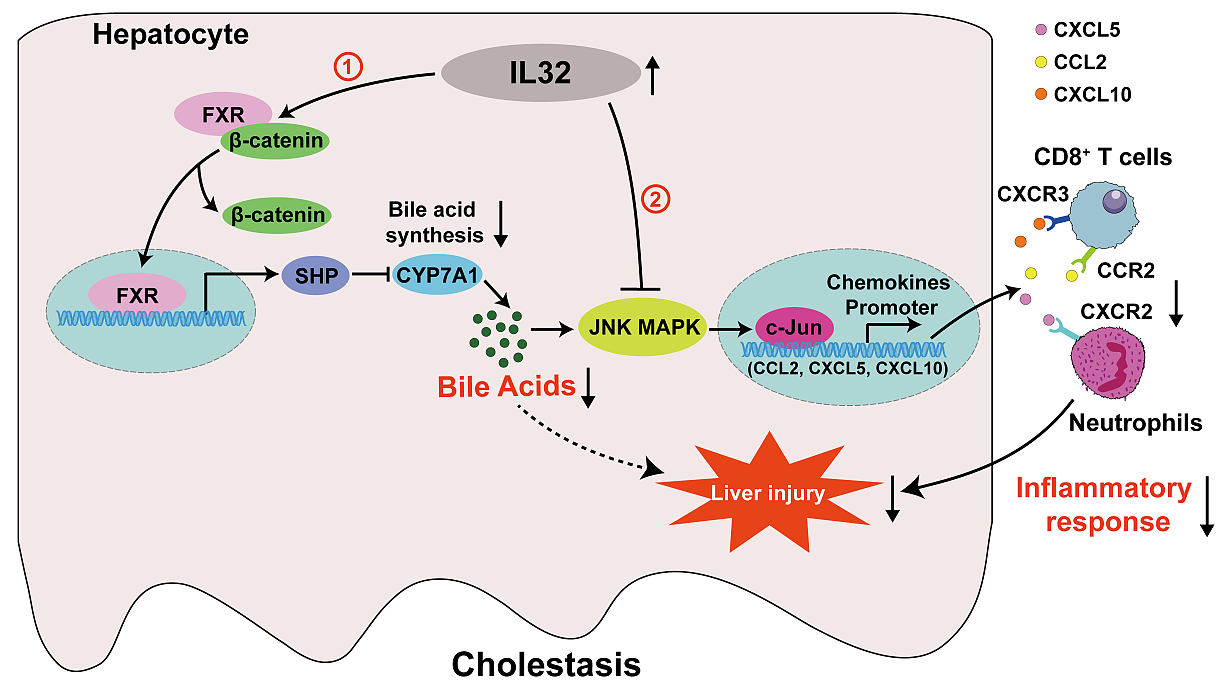


**Suppl. Figure 16.** Hepatic IL32 ameliorates cholestatic liver injury by repressing the accumulation of intrahepatic bile acids and the liver inflammatory response.

**Suppl. Tables.1-6**

**Table.S1 Clinical Features of Patients**

| **Clinical Features** | **Control patients** | **Obstructive**  **Cholestatic patients** |
| --- | --- | --- |
| Total samples (Male/Female) | 22 (12/10) | 35 (21/14) |
| Age (years) | 52 ± 13 | 54 ± 12 |
| ALT (*IU/L*) | 49.7 ± 35.4 | 169.9 ± 142.5* |
| AST (*IU/L*) | 49.5 ± 36.1 | 149.7 ± 94.3* |
| ALP (*IU/L*) | 92.6 ± 44.5 | 545.5 ± 350.4* |
| GGT (*IU/L*) | 72.9 ± 79.6 | 769.7 ± 567.2* |
| TBA (*μmol/L*) | 4.1 ± 3.4 | 94.9 ± 99.6* |
| TBIL (*μmol/L*) | 15.9 ± 8.8 | 186.0 ± 152.5* |
| DBIL (*μmol/L*) | 3.3 ± 1.9 | 94.9 ± 78.4* |
| IBIL (*μmol/L*) | 12.6 ± 7.8 | 91.2 ± 76.6* |

Values are means ± SD.

**Abbreviations:** ALT, alanine aminotransferase; AST, aspartate aminotransferase; ALP, alkaline phosphatase; GGT, gamma-glutamyl transferase; TBA, total bile salts; TBIL, total bilirubin; DBIL, direct bilirubin; IBIL, indirect bilirubin.

**P < 0.001*.

†*P < 0.01* versus controls.

**Table.S2 Serum biochemistry and liver tissue bile acid in BDL mice for 3 days**

|  | **WT-Sham**  **(n = 7)** | ***hIL32γLTg*-Sham (n = 8)** | **WT-BDL**  **(n = 12)** | ***hIL32γLTg*-BDL**  **(n = 11)** |
| --- | --- | --- | --- | --- |
| Serum ALT *(IU/L)* | 24.50 ± 7.91 | 22.82 ± 6.16 | 347.67 ± 124.45*,# | 174.35 ± 80.37*,#,**§** |
| Serum AST *(IU/L)* | 92.07 ± 17.42 | 81.29 ± 15.56 | 601.27 ± 282.58***,**# | 274.27 ± 95.13*,#,**§** |
| Serum ALP *(IU/L)* | 90.76 ± 37.99 | 92.35 ± 53.30 | 334.90 ± 119.92***,**# | 226.95 ± 88.36*,#,**§** |
| Serum TBA *(μmol/L)* | 1.72 ± 0.85 | 3.33 ± 3.31 | 217.46 ± 73.07***,**# | 135.06 ± 63.61*,#,**§** |
| Serum TBIL *(μmol/L)* | 0.91 ± 0.62 | 0.92 ± 0.91 | 131.80 ± 43.21***,**# | 115.12 ± 76.49*,# |
| Serum DBIL *(μmol/L)* | 0.23±0.27 | 0.36±0.49 | 91.24 ± 35.94***,**# | 72.27 ± 59.04*,# |
| Liver tissue BAs *(μmol/kg of liver)* | 110.99 ± 86.30 | 92.75 ± 48.23 | 346.90 ± 124.10*,# | 199.59 ± 106.64#,**§** |
| Liver 7-α-C4 (*ng/g of liver*) | Not detected | Not detected | 53.55 ± 10.69 | 29.73 ± 12.29**§** |

Values are means ± SD. ******P <* 0.05 versus WT-Sham mice; **#***P <* 0.05 versus hIL32γLTg-Sham mice; **§***P <* 0.05 versus WT-BDL mice.

**Abbreviations:** Sham, sham operation; BDL, bile duct-ligated operation; WT, wild type; *hIL32γLTg*, human *IL32γ* liver-specific transgenic mice; ALT, alanine aminotransferase; AST, aspartate aminotransferase; ALP, alkaline phosphatase; TBA, total bile salts; TBIL, total bilirubin; DBIL, direct bilirubin; BAs, bile acids.

**Table.S3 Serum biochemistry in mice fed with 1% CA for 14 days**

|  | **Chow Diet, 14 Days** | |  |  | **1% CA Diet, 14 Days** | | | |
| --- | --- | --- | --- | --- | --- | --- | --- | --- |
|  | **WT (n = 6)** | ***hIL32γLTg* (n = 5)** |  |  | **WT (n = 7)** | | ***hIL32γLTg* (n = 9)** | |
| Serum ALT (*IU/L*) | 29.24 ± 3.72 | 13.34 ± 5.66* | | 201.08 ± 94.29*,# | | 103.53 ± 57.80*,#,**§** | |  |
| Serum AST (*IU/L*) | 94.06 ± 47.74 | 87.53 ± 53.66 | | 212.27 ± 104.00***,**# | | 116.04 ± 57.12**§** | |  |
| Serum ALP (*IU/L*) | 68.82 ± 21.73 | 82.59 ± 38.79 | | 118.49 ± 57.34 | | 152.94 ± 40.74*,# | |  |
| Serum TBA (*μmol/L*) | 1.45 ± 0.60 | 2.48 ± 0.59* | | 274.32 ± 107.87***,**# | | 269.96 ± 59.24*,# | |  |
| Serum TBIL (*μmol/L*) | 1.65 ± 1.24 | 2.33 ± 1.02 | | 10.08 ± 6.53***,**# | | 13.73 ± 4.56*,# | |  |
| Serum DBIL (*μmol/L*) | 0.60 ± 0.34 | 0.77 ± 0.45 | | 2.97 ± 1.91***,**# | | 5.37 ± 5.43 | |  |

Values are means ± SD.

******P <* 0.05 versus WT-Chow diet mice; **#***P <* 0.05 versus *hIL32γLTg*-Chow diet mice; **§***P <* 0.05 versus WT-1% CA fed.

**Abbreviations:** CA, cholic acid; WT, wild type; *hIL32γLTg*, human *IL32γ* liver-specific transgenic mice; ALT, alanine aminotransferase; AST, aspartate aminotransferase; ALP, alkaline phosphatase; TBA, total bile salts; TBIL, total bilirubin; DBIL, direct bilirubin.

**Table.S4 Real-Time Quantitative PCR Probes (TaqMan) and Primers (SYBR)**

|  |  |  |
| --- | --- | --- |
| Gene | Sequences (5’🡪3’) | Species/Source |
| *GAPDH* | Proprietary to ABI | Human/Hs02758991_g1 |
| *IL32* | Proprietary to ABI | Human/Hs00992441_m1 |
| *^1^IL32α* | Forward:5'-CTGAAGGCCCGAATGCACCA-3'  Reverse:5'-CCGTAGGACTTGTCACAAAA-3' | Human/Primers (SYBR)  NM_001012633.3 |
| *^2^IL32β* | Forward:5'-CAGTGGAGCTGGGTCATCTCA-3'  Reverse:5'-GGGCCTTCAGCTTCTTCATGTCATCA-3' | Human/Primers (SYBR)  NM_001012632.2 |
| *^2^IL32γ* | Forward:5'-AGGCCCGAATGGTAATGCT-3'  Reverse:5'-CCACAGTGTCCTCAGTGTCACA-3' | Human/Primers (SYBR)  NM_001369587.2 |
| *Gapdh* | Proprietary to ABI | Mouse/Mm99999915_g1 |
| *Mrp2 (Abcc2)* | Proprietary to ABI | Mouse/Mm00496899_m1 |
| *Mrp3 (Abcc3)* | Proprietary to ABI | Mouse/Mm00551550_m1 |
| *Mrp4 (Abcc4)* | Proprietary to ABI | Mouse/Mm01226381_m1 |
| *Ostα (Slc51a)* | Proprietary to ABI | Mouse/Mm00521530_m1 |
| *Ostβ (Slc51b)* | Proprietary to ABI | Mouse/Mm01175040_m1 |
| *Bsep (Abcb11)* | Proprietary to ABI | Mouse/ Mm00445168_m1 |
| *Oatp1b2* *(Slco1b2)* | Proprietary to ABI | Mouse/ Mm00451510_m1 |
| *Cyp7a1* | Proprietary to ABI | Mouse/Mm00484150_m1 |
| *Cyp7b1* | Proprietary to ABI | Mouse/Mm00484157_m1 |
| *Cyp27a1* | Proprietary to ABI | Mouse/Mm00470430_m1 |
| *Cyp2b10* | Proprietary to ABI | Mouse/Mm01972453_s1 |
| *Ugt1a1* | Proprietary to ABI | Mouse/Mm02603337_m1 |
| *Sult2a1/2* | Proprietary to ABI | Mouse/Mm04205659_mH |
| *Fxr (Nr1h4)* | Proprietary to ABI | Mouse/Mm00436425_m1 |
| *Shp (Nr0b2)* | Proprietary to ABI | Mouse/Mm00442278_m1 |
| *Ccl2* | Proprietary to ABI | Mouse/Mm00441242_m1 |
| *Ccl5* | Proprietary to ABI | Mouse/Mm01302427_m1 |
| *Cxcl10* | Proprietary to ABI | Mouse/Mm00445235_m1 |
| *Tnfα* | Proprietary to ABI | Mouse/Mm00443258_m1 |
| *Il-1β* | Proprietary to ABI | Mouse/Mm00434228_m1 |
| *Il-6* | Proprietary to ABI | Mouse/Mm00446190_m1 |
| *α-SMA (Acta2)* | Proprietary to ABI | Mouse/Mm01546133_m1 |
| *Cola1* | Proprietary to ABI | Mouse/Mm00801666_g1 |
| *Cola2* | Proprietary to ABI | Mouse/Mm00483888_m1 |
| *Ck19 (Krt19)* | Proprietary to ABI | Mouse/Mm00492980_m1 |
| *Il-1α* | Forward:5'-aggagaagaccagcccgtgttgc-3'  Reverse:5'-tctgggatgggaggcaaataggg-3' | Mouse/Primers (SYBR)  XM_006498794.3 |
| *Cxcl1* | Forward: 5' -gaagggtgttgtgcgaaaagaag-3'  Reverse: 5' -cacaaaatgtccaagggaagcg-3' | Mouse/Primers (SYBR)  NM_008176.3 |
| *Cxcl2* | Forward: 5'-aggcatctgcttcggggactctggc-3'  Reverse: 5'-gcaaactcagccacaggggcgaagg-3' | Mouse/Primers (SYBR)  NM_015779.2 |
| *Cxcl5* | Forward: 5'-catttgatgtcttcgtagtatggca-3'  Reverse: 5'-ttccttgcttcttaccttcttcacc-3' | Mouse/Primers (SYBR)  [NM_009141.3](https://www.ncbi.nlm.nih.gov/entrez/viewer.fcgi?db=nucleotide&id=485464588) |
| *Cxcl7* | Forward: 5'-ctcagacctacatcgtcctgc-3'  Reverse: 5'-gtggctatcacttccacatcag-3' | Mouse/Primers (SYBR)  NM_023785.3 |
| *Cxcl9* | Forward: 5'-tccttttgggcatcatcttcc-3'  Reverse: 5'-tttgtagtggatcgtgcctcg-3' | Mouse/Primers (SYBR)  XM_021163528.1 |
| *Cxcl11* | Forward: 5'-ggcttccttatgttcaaacaggg-3'  Reverse: 5'-gccgttactcgggtaaattaca-3' | Mouse/Primers (SYBR)  NM_019494.1 |
| *Cxcl13* | Forward: 5'-ggccacggtattctggaagc-3'  Reverse: 5'-gggcgtaacttgaatccgatcta-3' | Mouse/Primers (SYBR)  XM_021163742.1 |
| *Cxcl16* | Forward: 5'-acccttgtctcttgcgttctt-3'  Reverse: 5'-caaagtaccctgcggtatctg-3' | Mouse/Primers (SYBR)  NM_023158.7 |
| *Ccl3* | Forward: 5'-tgtaccatgacactctgcaac-3'  Reverse: 5'-caacgatgaattggcgtggaa-3' | Mouse/Primers (SYBR)  NM_011337.2 |
| *Ccl4* | Forward: 5'-ttcctgctgtttctcttacacct-3'  Reverse: 5'-ctgtctgcctcttttggtcag-3' | Mouse/Primers (SYBR)  XM_011248832.1 |
| *Ccl7* | Forward: 5'-gctgctttcagcatccaagtg-3'  Reverse: 5'-ccagggacaccgactactg-3' | Mouse/Primers (SYBR)  NM_013654.3 |
| *Ccr2* | Forward: 5'-cctgtcatttatgcctttgttgga-3'  Reverse: 5'-tcacttactttacaacccaaccgag-3' | Mouse/Primers (SYBR)  [XM_006512428.2](https://www.ncbi.nlm.nih.gov/nucleotide/XM_006512428.2?report=genbank&log$=nuclalign&blast_rank=1&RID=EHWBDVAE014) |
| *Cxcr2* | Forward: 5'-gcacctgctctgtcaccgatgtct-3'  Reverse: 5'-agaatatcttgcacagggttgagcc-3' | Mouse/Primers (SYBR)  [XM_006495638.3](https://www.ncbi.nlm.nih.gov/nucleotide/XM_006495638.3?report=genbank&log$=nuclalign&blast_rank=1&RID=EHW5F1WA014) |
| *Cxcr3* | Forward: 5'-ggttagtgaacgtcaagtgct-3'  Reverse: 5'-ccccataatcgtagggagaggt-3' | Mouse/Primers (SYBR)  [NM_009910.3](https://www.ncbi.nlm.nih.gov/entrez/viewer.fcgi?db=nucleotide&id=818213420) |
| *Ptprc* | Forward: 5'-tgggaagattcaaagaaatgggact-3'  Reverse: 5'-gccattagtttcataaggaggacca-3' | Mouse/Primers (SYBR)  NM_001268286.1 |
| *Mpo* | Forward: 5'-tggttgcccgcagagtatga-3'  Reverse: 5'-tgaagaaggagaagcgggtag-3' | Mouse/Primers (SYBR)  [NM_010824.2](https://www.ncbi.nlm.nih.gov/entrez/viewer.fcgi?db=nucleotide&id=226823249) |
| *Cd4* | Forward: 5'-gccacggtttccctccctct-3'  Reverse: 5'-acaccagtcccaccatccg-3' | Mouse/Primers (SYBR)  NM_013488.3 |
| *Cd8a* | Forward: 5'-tgctgtcttatgtattgccctctg-3'  Reverse: 5'-gctctggtgttacagtctgctcatt-3' | Mouse/Primers (SYBR)  NM_009857.1 |
| *Fgfr4* | Forward: 5'-ttggccctgttgagcatcttt-3'  Reverse: 5'-gccctctttgtaccagtgacg-3' | Mouse/Primers (SYBR)  [NM_008011.2](https://www.ncbi.nlm.nih.gov/entrez/viewer.fcgi?db=nucleotide&id=112293259) |
| *β-catenin* | Forward: 5'-atggagccggacagaaaagc-3'  Reverse: 5'-cttgccactcagggaagga-3' | Mouse/Primers (SYBR)  [NM_001165902.1](https://www.ncbi.nlm.nih.gov/entrez/viewer.fcgi?db=nucleotide&id=260166641) |

**References:**

1. Jeong HJ, Shin SY, Oh HA, et al. IL-32 up-regulation is associated with inflammatory cytokine production in allergic rhinitis. *J Pathol.* 2011;224(4):553-563.
2. Plantinga TS, Costantini I, Heinhuis B, et al. A promoter polymorphism in human interleukin-32 modulates its expression and influences the risk and the outcome of epithelial cell-derived thyroid carcinoma. *Carcinogenesis*. 2013;34(7):1529-1535.

**Table.S5 Antibodies used in Western Blot, Chromatin Co-immunoprecipitation, Co-immunoprecipitation, and Immunohistochemistry**

|  |  |  |  |
| --- | --- | --- | --- |
| Protein | Host | Company / Catalog | Antibody dilution |
| GAPDH | Rabbit | Proteintech, Chicago, IL/10494-1-AP | WB 1:3000 |
| Lamin A | Rabbit | Abcam, Cambridge, MA/ab26300 | WB 1:1000 |
| IL32 | Rabbit | Proteintech, Chicago, IL/11079-1-AP | WB 1:2000; IHC 1:50 |
| SH-PTP1 (C-19) | Rabbit | Santa Cruz, Dallas, CA/sc-287 | WB 1:2500 |
| ABCB4 | Rabbit | Invitrogen, Carlsbad, CA/PA5-78692 | WB 1:2000 |
| OSTα (SLC51A) | Rabbit | Santa Cruz, Dallas, CA/sc-100078 | WB 1:2000 |
| OSTβ (SLC51B) | Rabbit | Sigma-Aldrich, St Louis, MO/HPA008533 | WB 1:500 |
| FXR (NR1H4) | Rabbit | Proteintech, Chicago, IL/25055-1-AP | WB 1:2000; IHC 1:200; CO-IP 2μg per sample; ChIP 2μg per sample |
| SHP (NR0B2) | Rabbit | Abcam, Cambridge, MA/ab96605 | WB 1:1000 |
| β-catenin | Rabbit | Proteintech, Chicago, IL/51067-2-AP | WB 1:4000 |
| CYP7A1 (H-58) | Rabbit | Santa Cruz, Dallas, CA/sc-25536 | WB 1:1000 |
| Phoshpo-ERK1/2 (Thr202/Tyr204) (D13.14.4E) | Rabbit | Cell Signaling, Beverly, MA/#4370 | WB 1:2500 |
| ERK1/2 | Rabbit | Cell Signaling, Beverly, MA/#9102 | WB 1:2500 |
| Phospho-JNK (Thr183/Tyr185) (G9) | Mouse | Cell Signaling, Beverly, MA/#9255 | WB 1:2000 |
| JNK (D-2) | Mouse | Santa Cruz, Dallas, CA/sc-7345 | WB 1:1000 |
| c-Jun (E254) | Rabbit | Abcam, Cambridge, MA/ab32137 | WB 1:2000 |
| c-Jun (60A8) | Rabbit | Cell Signaling, Beverly, MA/#9165 | ChIP 2μg per sample |
| FGF19 | Rabbit | Abcam, Cambridge, MA/ab225942 | WB 1:2000 |
| FGFR4 | Rabbit | Proteintech, Chicago, IL/11098-1-AP | WB 1:2000 |
| CCL2 | Rabbit | ProSci, Poway, CA/7201 | WB 1:2000 |
| CXCL5 | Rabbit | Abcam, Cambridge, MA/ab9802 | WB 1:2000 |
| CXCL10 | Rabbit | Biorbyt, Cambridge, UK/orb10277 | WB 1:2000 |
| Myeloperoxidase | Rabbit | Abcam, Cambridge, MA/ab188211 | WB 1:1000 |
| CD8α (D4W2Z) | Rabbit | Cell Signaling, Beverly, MA/#98941 | WB 1:1000 |
| CD8a | Mouse | Proteintech, Chicago, IL/66868-1-Ig | WB 1:1000 |
| Cytokeratin 19 (CK19) | Rabbit | Abcam, Cambridge, MA/ab15463 | IHC 1:400 |

**Table.S6A PCR primers for ChIP assays in mouse liver (Real time qPCR and semi-quantity PCR)**

| ChIP | Primer pairs | Products (bp) |
| --- | --- | --- |
| Fxr ChIP  (Shp Site-1139/-1129) | Forward: 5’- CTCCCAGGAGTGGCTAGAA-3’  Reverse: 5’- GCATCACCCTGGATAAGTGATGG-3’ | 134 bp |
| c-Jun ChIP1 (Cxcl5 Site-1711/-1705) | Forward: 5’- GCTGCTTGTTACAGATTACCACT -3’  Reverse: 5’- TGATCGCATTGTGAGAAGCTAA -3’ | 114 bp |
| c-Jun ChIP2 (Cxcl5 Site-1157/-1151) | Forward: 5’- AGAACGGATGCTATCTTGCT -3’  Reverse: 5’- CCTGCCAGATAGGGATTTGACA -3’ | 134 bp |
| c-Jun ChIP3 (Cxcl5 Site-924/-918) | Forward: 5’- GACAGACTGCTCCTAACTGA -3’  Reverse: 5’- CACTTAAACCAGCTGCATGA -3’ | 169 bp |
| c-JunChIP4 (Cxcl10 site-765/-759) | Forward: 5’- TGTTTCCCAGTTCCCGTTG -3’  Reverse: 5’- ATAGATCTCGCATAGAGGT -3’ | 147 bp |
| c-Jun ChIP5 (Cxcl10 site-74/-68) | Forward: 5’- TTTCTCAAACAGCTCACGCTT -3’  Reverse: 5’- GCTGACTTTGGAGATGACT -3’ | 172bp |
| c-Jun ChIP6 (Ccl2 site -21/-15) | Forward: 5’- CTGCACTTACTCAGCGGAT -3’  Reverse: 5’- CTGGCCTTCCTCCCGTCTG -3’ | 163 bp |
| ChIP for positive control (Gapdh) | Forward: 5’-CCTCTGCGCCCTTGAGCTAGGA-3’  Reverse: 5’-CACAAGAAGATGCGGCCGTCTC-3’ | 166 bp |

**Table.S6B PCR primers for ChIP assays in human liver (Real time qPCR and semi-quantity PCR)**

| ChIP | Primer pairs | | Products (bp) |
| --- | --- | --- | --- |
| FXR ChIP  (SHP site -1894/-1884) | Forward: 5’- GCCCCATAAAACCAATATCCA -3’  Reverse: 5’- ATCCTGCTTCACTTAGCTG-3’ | 119bp | |
| c-Jun ChIP1  (CCL2 site -726/-720) | Forward: 5’- ACCCAAGCAGGCAACTAGTTG -3’  Reverse: 5’- AACTCTGTAAATTCCCCAACT -3’ | | 175 bp |
| c-Jun ChIP2  (CXCL5 site-278/-272) | Forward: 5’- GCTCCTGTTACTTTGGTTCCAC -3’  Reverse: 5’- GACGTTGTAACCCTGCTCCG -3’ | | 199 bp |
| c-Jun ChIP3  (CXCL10 site -989/-983) | Forward: 5’- TCATGTGCTTTCTACTGCT -3’  Reverse: 5’- TTGGCACAATTTTAACTGGA -3’ | | 106 bp |
| ChIP for positive control (GAPDH) | Forward: 5’-TACTAGCGGTTTTACGGGCG -3’  Reverse: 5’-TCGAACAGGAGGAGCAGAGAGCGA -3’ | | 166 bp |

**Table.S6C TFs binding sites at Mus promoters**

| TFs | Promoter sites | Predicted binding site sequence |
| --- | --- | --- |
| Fxr | Shp (-1139/-1129) | CCAAGGTCAGG |
| c-Jun | Ccl2 (-21/-15) | TGCCTCA |
| c-Jun | Cxcl5(-924/-918) | TGAGTCA |
| c-Jun | Cxcl5(-1157/-1151) | TGAATCA |
| c-Jun | Cxcl5(-1711/-1705) | TGAGTCA |
| c-Jun | Cxcl10(-74/-68) | TGACTCA |
| c-Jun | Cxcl10(-765/-759) | TGACTCA |

**Table.S6D TFs binding sites at Homo promoters**

| TFs | Promoter sites | Predicted binding site sequence |
| --- | --- | --- |
| FXR | SHP (-1894/-1884) | CCAAGGTCACT |
| c-Jun | CCL2 (-726/-720) | TGCCTCA |
| c-Jun | CXCL5(-1633/-1627) | TGACTCA |
| c-Jun | CXCL5(-278/-272) | TGCCTCA |
| c-Jun | CXCL10(-989/-983) | TGACTGA |
